# Within- and trans-generational plasticity: seed germination responses to light quantity and quality

**DOI:** 10.1093/aobpla/ply023

**Published:** 2018-04-10

**Authors:** Katherine Vayda, Kathleen Donohue, Gabriela Alejandra Auge

**Affiliations:** Department of Biology, Duke University, Durham, NC, USA

**Keywords:** Dormancy, germination, light, maternal effects, plasticity, secondary dormancy

## Abstract

Plants respond not only to the environment in which they find themselves, but also to that of their parents. The combination of within- and trans-generational phenotypic plasticity regulates plant development. Plants use light as source of energy and also as a cue of competitive conditions, since the quality of light (ratio of red to far-red light, R:FR) indicates the presence of neighbouring plants. Light regulates many aspects of plant development, including seed germination. To understand how seeds integrate environmental cues experienced at different times, we quantified germination responses to changes in light quantity (irradiance) and quality (R:FR) experienced during seed maturation and seed imbibition in *Arabidopsis thaliana* genotypes that differ in their innate dormancy levels and after treatments that break or reinduce dormancy. In two of the genotypes tested, reduced irradiance as well as reduced R:FR during seed maturation induced higher germination; thus, the responses to light quantity and R:FR reinforced each other. In contrast, in a third genotype, reduced irradiance during seed maturation induced progeny germination, but response to reduced R:FR was in the opposite direction, leading to a very weak or no overall effect of a simulated canopy experienced by the mother plant. During seed imbibition, reduced irradiance and reduced R:FR caused lower germination in all genotypes. Therefore, responses to light experienced at different times (maturation vs. imbibition) can have opposite effects. In summary, seeds responded both to light resources (irradiance) and to cues of competition (R:FR), and trans-generational plasticity to light frequently opposed and was stronger than within-generation plasticity.

## Teaser

The ability to predict potential competitive environments allows plants to adjust to conditions that could limit growth and survival. Here we show that seeds integrate information from light environments experienced by mother plants and by seed themselves. Seeds responded to distinct components of the light environment: light wavelength, which predicts the presence of neighbours, and light quantity, which indicates resource abundance. Responses to the maternal environment were stronger than responses to the seed environment, suggesting that the integration of environmental information across generations may have different predictive value depending on the generation that experienced the cue.

## Introduction

Plants respond not only to the environmental conditions in which they find themselves (within-generational plasticity), but also to those of their parents (trans-generational plasticity) (Schlichting 1986; Kirkpatrick and Lande 1989; Sultan 2000; Galloway and Etterson 2007; Snell-Rood 2013; reviewed in Auge *et al.* 2017b). When dispersal is limited and environments are stable over time, parental environments may be accurate predictors of progeny environments, such that trans-generational plasticity can induce progeny phenotypes that are suited to their environments (Galloway and Etterson 2007; Holeski *et al.* 2012; Latzel *et al.* 2014; Dey *et al.* 2016; Herman and Sultan 2016; Singh *et al.* 2017). Alternatively, when environments vary over time, parental environments may be less accurate predictors of future selective conditions (Ezard *et al.* 2014; English *et al.* 2015), and plastic responses to the individual’s own environment may be more adaptive than responses to parental environments. However, when environments vary predictably or cyclically, such as occurs with seasonal variation, parental environments may be more accurate predictors of progeny’s future environments than the progeny’s own environment (Ezard *et al.* 2014; Prizak *et al.* 2014; Kuijper and Hoyle 2015). How within- and trans-generational plasticity combine to regulate progeny phenotypes is important for understanding development under temporally variable conditions (Auge *et al.* 2017b).

Seed germination is an important developmental event in plants. It must be timed so that it occurs only under environmental conditions that permit growth and survival (Donohue *et al.* 2005; Graeber *et al.* 2012; Baskin and Baskin 2014). As such, germination is highly environmentally cued, and it responds to environmental conditions experienced both by maternal parents and by seeds themselves (Gutterman 2000; Donohue 2009; Graeber *et al.* 2012). In *Arabidopsis thaliana*, for example, chilling of maternal parents even before the initiation of reproduction increases germination of the progeny, while cooler temperatures and longer days during reproduction decrease germination (Chen *et al.* 2014; Auge *et al.* 2017a; Imaizumi *et al.* 2017). Some environmental factors experienced in parental generations can alter how seeds respond to their own environments (reviewed in Auge *et al.* 2017b).

Dormancy—a block to germination in conditions otherwise favourable for germination—is expected to influence germination responses to many environmental cues, and it may even be necessary for the regulation of germination by certain maternal environments. Dormancy varies genetically, with extensive ecotypic variation observed in *A. thaliana* (Kronholm *et al.* 2012; Debieu *et al.* 2013)—genotypes can differ in their ability to induce and maintain dormancy, or in their rate of dormancy loss over time. How genetically determined dormancy and maternal effects interact to regulate germination timing is largely unexplored.

Parental and progeny environments frequently combine to influence progeny germination (Auge *et al.* 2017b). Within- and trans-generational plasticity might influence germination in different ways: (i) acting additively and in the same direction (Lacey 1996; Bernareggi *et al.* 2016); (ii) operating in opposite directions (Leverett *et al.* 2016); or (iii) overriding responses to each other (within-generation cues overriding the maternal environment: Vu *et al.* 2015; Groot *et al.* 2016; Moriuchi *et al.* 2016; or parental cues overriding the progeny environment: Zhang *et al.* 2012; Vu *et al.* 2015; Leverett *et al.* 2016; Moriuchi *et al.* 2016). Germination responses to light environment is a recently documented example in which responses to parental environments can be stronger than responses to the progeny’s own environment (Leverett *et al.* 2016).

Light conditions experienced by a plant provide cues of burial, shading or the presence of neighbouring vegetation (Casal and Sánchez 1998). The presence of neutral shading (imposed by a fixed structure) or a vegetation canopy may pose risks to a plant’s survival and growth, as they will limit a plant’s access to light. Therefore, postponing germination until light conditions are favourable may be advantageous, as long as shaded conditions are temporary (Deregibus *et al.* 1994; Dechaine *et al.* 2009; Leverett *et al.* 2018). However, in the presence of a vegetation canopy—indicating potential future competition with plant neighbours for light and other resources—early germination could provide a head-start and a competitive advantage that allows the plant to pre-empt or outgrow its competitors (Schmitt 1997). Therefore, light quantity and light quality could provide important cues about immediate and future resources, and seeds may postpone or accelerate their germination in response to them, potentially improving their chances of establishment and growth.

Distinguishing plastic responses to resources alone vs. cues of future environments is relevant for interpreting conditions under which plasticity is adaptive as opposed to a ‘passive’ response to resource limitation (Schlichting and Pigliucci 1998; Dorn *et al.* 2000). Phenotypic plasticity may be caused by resource limitation if phenotypes are costly to induce or maintain. ‘Active’ responses to cues have been hypothesized more likely to be adaptive in the environments under which they evolved (Schlichting and Pigliucci 1998). For traits that are responsive to environments experienced both by parents and by progeny, it is particularly interesting to evaluate whether resource limitation in parental vs. progeny generations more strongly influences offspring traits. Such an evaluation can provide insight into how parent–progeny interactions, including parent–offspring conflicts, imposed by resource limitation may compromise adaptive progeny phenotypes, or alternatively whether adaptive progeny responses induced by parents can ameliorate resource limitation of vulnerable progeny. Such studies are especially interesting in cases in which responses to paternal and progeny environments oppose each other, as in the case of progeny responses to parental vs. progeny light environments. In a recent study (Leverett *et al.* 2016), a simulated vegetative canopy (green filter) experienced by maternal plants increased seed germination, but seed imbibition under the same conditions either decreased or had no effect on progeny germination. Whether these responses were mediated by resource limitation or by responding to R:FR cues of competition is not known, since the simulated canopy used in the study reduced both total irradiance and R:FR. In addition, the extent to which germination responses to these light cues depend on the level of seed dormancy is not known. Insight into how seeds respond to canopy environments that change throughout a season requires investigations of how the responses of seeds to light depend on dynamically changing dormancy levels.

This study aims to quantify within- and trans-generational plasticity of germination of *A. thaliana* seeds in response to a simulated vegetation canopy, and to identify the degree to which these germination responses are caused by responses to light limitation (irradiance) as opposed to cues of competition (R:FR). We compared the germination of genotypes that differ in dormancy and that have experienced different treatments to relieve or induce dormancy. Specifically, we asked: (i) To what extent is the observed response to vegetation canopy caused by responses to reduced irradiance or reduced R:FR? (ii) What is the direction and magnitude of germination responses to light quantity and quality experienced by maternal parents vs. imbibing seeds? (iii) How are germination responses to light environments during maturation and imbibition influenced by the depth of seed dormancy?

## Methods

We manipulated the light environment experienced during the maturation and imbibition of *A. thaliana* seeds. The light treatments used during maturation were: a clear filter as a control white-light environment (WL), a neutral filter to simulate neutral shade/reduced irradiance (NF), and a green filter to simulate a vegetation canopy with reduced irradiance and reduced R:FR (GF). Dormancy was manipulated by using eight different genotypes that differed in dormancy. To establish different dormancy levels in each genotype, those seeds were assayed fresh, were allowed to after-ripen and lose primary dormancy, or were induced into secondary dormancy. Seeds were then imbibed under the same three light treatments as during maturation (WL, NF, GF), as well as at two different incubation temperatures. **Figure S1** shows an outline of the different treatments employed **[see****Supporting Information—Fig. S1****]**.

### Study species

We used *A. thaliana* (Brassicaceae) for this experiment. *Arabidopsis thaliana* is typically a winter annual in its native range, germinating in autumn and flowering in the spring (Baskin and Baskin 2014). It also can display a rapid cycling behaviour (Ratcliffe 1976; Donohue 2009). Such variation in life-history variation can be attributed, in part, to variability in germination and dormancy (Baskin and Baskin 2014). The seeds of *A. thaliana* have physiological dormancy (Baskin and Baskin 2014), requiring a chemical change within the seeds for them to germinate. Dormancy varies among *A. thaliana* populations (Montesinos-Navarro *et al.* 2012; Debieu *et al.* 2013; Postma *et al.* 2016) and such variation has been demonstrated to be the target of natural selection and contribute to local adaptation (Kronholm *et al.* 2012).

### Genetic material

Natural allelic variation has been documented in genes that influence dormancy, including the germination-promoter gene *FLOWERING LOCUS C* (*FLC*; Chiang *et al.* 2009; Blair *et al.* 2017), which is up-regulated by the gene *FRIGIDA* (*FRI*; Clarke and Dean 1994; Koornneef *et al.* 1994; Blair *et al.* 2017). To determine whether seed dormancy affects germination responses to reduced irradiance and reduced R:FR, we tested two different backgrounds of *A. thaliana*: Landsberg *erecta* (L*er*, more dormant) and Columbia (Col, less dormant) (Chiang *et al.* 2009; Burghardt *et al.* 2016). Within each of these two backgrounds, we used different genetic variants in order to test whether activity levels of the gene *FLC* altered germination responses to light conditions. For L*er*, these genotypes included L*er* (with naturally weak *FLC*), the near isogenic line L*er*-*FLC* (Alonso-Blanco *et al.* 1998) and two RNAi::*FLC* lines (RNAi #1 and RNAi #2). In L*er*-*FLC*, a chromosomal region containing the *FLC* allele from the Cape Verde Island (Cvi) ecotype was introgressed into the L*er* background. This introgressed locus has higher expression of *FLC* than the L*er* background. In the two RNAi lines, RNA interference was used to knock down the expression of *FLC* in the L*er-FLC* line (Blair *et al.* 2017), allowing us to determine whether *FLC* influences germination responses to light. Thus, L*er* had intermediate dormancy, and L*er*-*FLC* had lower dormancy; the comparison of L*er*-*FLC* and the RNAi lines tested whether *FLC* causally alters germination responses to light.

Within the Columbia (Col) genotype, the four genotypes, each with different combinations of the active and inactive *FRIGIDA* and *FLC* genes, were studied. The *FRIGIDA* gene is known to up-regulate *FLC*, and thus inactive *FRIGIDA* (*FRI*) results in reduced *FLC* expression (Clarke and Dean 1994; Koornneef *et al.* 1994). Col has a naturally non-functional *FRI* allele and therefore low levels of *FLC* expression. Col-*FRI*_Sf_, on the other hand, has a functional *FRI* allele introgressed from the San Feliu ecotype and thus the potential to exhibit high levels of *FLC* expression. In addition, we used a knockout mutation of *FLC*: *flc*-3. We used all combination of functional and non-functional *FRI* and *FLC* alleles, specifically: Col (fri/FLC), Col *flc*-3 (fri/flc), Col-*FRI*_sf_ (FRI/FLC) and finally Col-*FRI*_sf_-*flc*-3 (FRI/flc). Examining the differences in germination response between different genotypes and backgrounds allowed us to discern how light environment alters the effects of genetic differences, particularly the disruption of the genes *FRIGIDA* and *FLC*. **Table S1** contains a summary of the eight genotypes **[see****Supporting Information—Table S1****]**. Seeds were kindly supplied by Scott Michaels and Maarten Koornneef, or purchased from the Arabidopsis Seed Stock Center at the Ohio State University.

Seeds for the experiments (maternal generation) were obtained by growing grandparental plants in standardized conditions as follows: seeds from all genotypes were potted in Metromix 360 soil (Scotts Sierra, Maysville, OH, USA) and pots were placed in GCW-30 growth chambers (Environmental Growth Chambers, Chagrin Falls, OH, USA) at 22 °C with a 12-h light cycle for plants to grow until bolting. Then, plants were moved to an 8-h light cycle at 15 °C until harvest. Seeds were kept in dry conditions at room temperature until used for the experiment.

### Maternal plant growth conditions

We potted seeds from all eight genotypes in Metromix 360 soil (Scotts Sierra, Maysville, OH, USA) where they then germinated and grew for 1 week. To induce and to ensure that flowering was synchronized among the plants, we vernalized these seedlings for 4 weeks at 4 °C. After vernalization, plants were moved to GCW-30 growth chambers (Environmental Growth Chambers, Chagrin Falls, OH, USA) where they grew at 22 °C with a 12-h light cycle. Once the plants began to bolt, plants were transferred to GCW-30 growth chambers at 15 °C with an 8-h light cycle and fertilized once with a 300 ppm nitrogen solution of Blossom Booster Fertilizer (JR Peters, Allentown, PA, USA). At this point, 12 biological replicates of each genotype were placed under three different light filters (*LEE* Filters, Andover, Hampshire, UK), thus establishing three maturation light environments. The maturation environments were: clear filter, neutral filter and green filter (#130 Clear, #298 0.15ND and #730 Liberty Green, respectively). The clear filter (‘WL’ or white light), used as the control, had an irradiance value of 290 μmol m^−2^ s^−1^ and an R:FR of 1.58. The neutral filter (‘NF’), used to reduce irradiance, had a reduced total irradiance of 200 μmol m^−2^ s^−1^, while the R:FR remained relatively high at 1.3. Lastly, the green filter (‘GF’), used to reduce both irradiance and R:FR, had a total irradiance of 160 μmol m^−2^ s^−1^ and reduced R:FR to 0.86. Even though the manufacturer’s information indicated that transmittance of the neutral and green filters was similar (69.3 and 67.5 %, respectively), we observed a more pronounced reduction in total irradiance of the green filter in our conditions. We address this issue when discussing the comparisons in the Discussion section. Germination responses to reduced irradiance (quantity) are revealed by comparing WL to NF; germination responses to reduced R:FR are revealed by comparing NF to GF—with the caveat that the green filter also reduced irradiance slightly compared to NF, which could confound results.

Due to differences in seed development and maturation timing, seeds were harvested in three batches: (i) all four genotypes in the L*er* background matured under GF, (ii) all four genotypes in the L*er* background matured under WL and NF (2 weeks later than the first batch) and (iii) all four genotypes in Col background matured under the three filters. For the L*er* background, the comparisons with the maternal GF treatment could be confounded with batch effects, although all conditions other than light were controlled across batches. This batch effect unlikely to account for responses to low maternal R:FR, since our observed effect of the maternal environment is consistent with that observed in previous studies of the same genotypes (Leverett *et al.* 2016).

### Germination assays

Fresh seeds (‘Fresh’: 2 days after harvest), after-ripened seeds (‘AR’: 5 months after harvest) and after-ripened seeds induced into secondary dormancy (‘SD’: 5-months after-ripened seeds that were subsequently incubated in the dark at 35 °C) (Auge *et al.* 2015; Leverett *et al.* 2016) were tested to examine how different dormancy levels affected germination response to reduced irradiance and reduced R:FR. Seeds were incubated under the same three light filters during imbibition as used during the maturation of the seeds—clear, neutral and green filters—in addition to a fourth light condition: darkness. The dark treatment served to determine whether light was required for the seeds to germinate, while we used the three filter conditions to examine the effects of light quantity or quality during imbibition on germination response. Twelve biological replicates (different mother plants from a single batch) for each genotype and maturation light treatment were incubated in each of the four imbibition light treatments. Seeds were assayed at two temperatures—10 and 22 °C—because temperature is known to influence the intensity of responses to maternal or seed environment effects (Auge *et al.* 2015; Burghardt *et al.* 2016; Leverett *et al.* 2016). We sowed 20 seeds per each of the 12 biological replicates for the eight genotypes in Petri plates on 0.6 % (w/v) agar. To prevent the plates from desiccating, we wrapped each one in Parafilm and immediately placed them directly into their light and temperature treatments in GC-82 growth chambers (Environmental Growth Chambers, Chagrin Falls, OH, USA) with a 12-h photoperiod. We placed the plates undergoing treatment with the clear, neutral and green filters on plastic trays. We wrapped the bottoms of these trays in aluminium foil and outfitted each lid with one of the three filters to ensure the seeds experienced only their specific imbibition light treatment. We randomly arranged the plates within each of the trays and we regularly randomized the position of the trays within the incubators. Plates with seeds undergoing the dark treatment during imbibition were placed into cardboard boxes that we then wrapped with two layers of aluminium foil. We censused plates under the clear, neutral and green filter treatments every week for 4 weeks, at which point germination plateaued for all seeds within the experiment. Seeds in dark-treatment plates were censused only at the end of the experiment, 4 weeks after the start of imbibition. To quantify germination response, we recorded the germination proportion for each individual plate—the number of germinated seeds (when radicle emerged from the seed coat) per total number of viable seeds. A seed was considered to be viable if they remained firm after germination plateaued.

### Statistical analysis

All statistical analyses were conducted using R v.3.3.1 (R Core Team 2017). Analyses presented in the main text used the three focal genotypes: L*er*, L*er-FLC* and Col; analyses of direct genetic manipulation of *FLC* are presented as supplementary material **[see****Supporting Information—Supplementary Text****]**. To test for effects of maternal treatment (Mat) on germination, and to test how that effect differed among genotypes (Geno), imbibition light treatments (Imbibe), imbibition temperatures (Temp) and dormancy treatments (after-ripening, ‘AR’, or secondary-dormancy induction, ‘SD’), we fit generalized linear models with logit link functions using ‘glm’, and then performed type-III likelihood ratio tests using the ‘Anova’ function in the ‘car’ package (Fox and Weisberg 2010). A logit link function was used because germination is a binomial trait, and the dependent variable was therefore in the form of proportions. A correction for multiple comparisons was conducted when appropriate, using the ‘Holm’ method of ‘p.adjust’ in ‘stats’ package. The dark incubation treatment was removed from the full model, as germination in the dark was uniformly low and lacked variance across treatments.

A full model that included Mat, Imbibe (minus dark treatment), Geno, Temp and dormancy treatments (Fresh, AR and SD) could not converge, so after-ripened seeds induced into secondary dormancy (SD), which had low germination proportions and low variance among treatments, were removed from the full model (Table 1). Genotype effects (specifically Col vs. the others) and Mat effects in L*er* (specifically GF vs. the others) could be confounded with batch, so Table 1 should be interpreted with caution. Nonetheless, and as mentioned above, our results are consistent with the results observed in previous studies (Leverett *et al.* 2016). All factors were considered as fixed effects. Because of significant interactions with genotype, submodels were used to analyse significant interactions between Temp, Mat, Imbibe, AR (Fresh vs. AR) and SD (AR vs. SD) for the three genotypes separately (L*er*, L*er*-*FLC* and Col). To interpret the significant interactions from the full model and submodels, pairwise contrasts between maternal conditions and imbibition treatments were performed **[see****Supporting Information—Table S2****]**.

**Table 1.** Result of full model to test for effects of genotype (‘Geno’: L*er*, L*er*-*FLC* and Col), temperature (‘Temp’: 10 and 22 °C), maternal light treatment (‘Mat’), seed imbibition light treatment (‘Imbibe’), after-ripening treatment (‘AR’: Fresh and AR) and their interactions, on germination proportions. Dark-imbibed seeds and seeds induced into secondary dormancy were dropped from the analysis because of low variance; therefore, ‘AR’ refers to the comparison between Fresh and AR seeds. Analyses are based on logit-linked generalized linear models. Likelihood ratios were tested based on chi-squares. Reference levels were L*er* (Geno), 10 °C (Temp), NF (Mat), NF (Imbibe) and fresh seeds (AR). Significance is expressed as *P < 0.05, **P < 0.01, ***P < 0.001.

Source of variation	df	LR chi-sq
Geno	2	6.52*
Temp	1	2.35
Mat	2	62.41***
Imbibe	2	1.15
AR	1	6.61**
Geno × Temp	2	14.05***
Geno × Mat	4	131.69***
Temp × Mat	2	10.75**
Geno × Imbibe	4	12.42*
Temp × Imbibe	2	12.68**
Mat × Imbibe	4	4.23
Geno × AR	2	27.10***
Temp × AR	1	1.95
Mat × AR	2	24.35***
Imbibe × AR	2	49.90***
Geno × Temp × Mat	4	51.95***
Geno × Temp × Imbibe	4	28.98***
Geno × Mat × Imbibe	8	9.95
Temp × Mat × Imbibe	4	4.06
Geno × Temp × AR	2	3.00
Geno × Mat × AR	4	37.14***
Temp × Mat × AR	2	26.90***
Geno × Imbibe × AR	4	34.02***
Temp × Imbibe × AR	2	1.51
Mat × Imbibe × AR	4	12.43*
Geno × Temp × Mat × Imbibe	8	16.56*
Geno × Temp × Mat × AR	4	17.83**
Geno × Temp × Imbibe × AR	4	8.72
Geno × Mat × Imbibe × AR	8	20.34*
Temp × Mat × Imbibe × AR	4	2.67
Geno × Temp × Mat × Imbibe × AR	8	11.78

To examine how *FLC* activity influenced germination response to light, each background was analysed separately [Supporting Information—Supplementary text; Supporting Information—Tables S3and S4; Supporting Information—Figures S6and S7]. Specifically, in the L*er* background, the two RNAi knock-downs of *FLC* were compared to L*er*-*FLC* to assess the effect on progeny germination of functional and non-functional *FLC*. In the Col background, the effect of disrupting *FLC* on functional and non-functional *FRI* was also investigated. Capital letters indicate functional alleles and lower case indicates non-functional alleles as follows: Col (fri/FLC) was compared to Col-*flc*-3 (fri/flc) to compare active vs. non-active *FLC* on a non-functional *FRI* background. Col-*FRI*_sf_ (FRI/FLC) was compared to Col-*FRI*_sf_-*flc*-3 (FRI/flc) to compare active vs. non-active *FLC* on a functional *FRI* background. Col (fri/FLC) was compared to Col-*FRI*_sf_ (FRI/FLC) to compare active vs. non-active *FRI* on a functional *FLC* background. Finally, Col-*flc*-3 (fri/flc) was compared to Col-*FRI*_sf_-*flc*-3 (FRI/flc) to compare active vs. non-active *FRI* on a non-functional *FLC* background.

## Results

**Figure 1.**
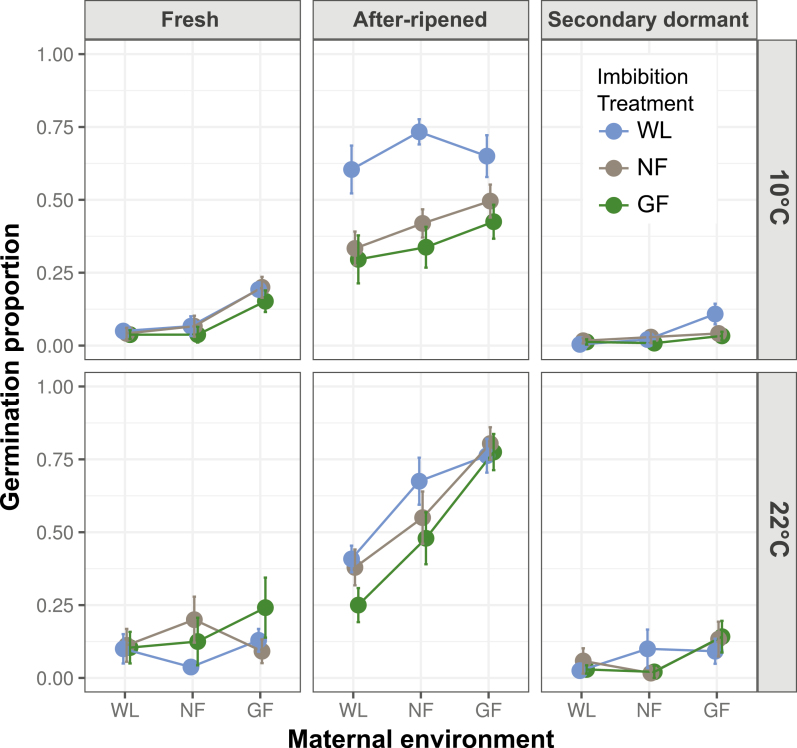
Effect of maturation under white light (WL), a neutral filter (NF) and a green filter (GF) (*x*-axis) on germination of fresh seeds, after-ripened seeds (AR) and seeds induced into secondary dormancy (SD) of the L*er* genotype incubated under WL, NF or GF (see key), and at either 10 °C (upper panel) or 22 °C (lower panel). For statistical significance of pairwise comparisons (between maternal and seed imbibition conditions), see **Supporting Information—Table S2**.

**Figure 2.**
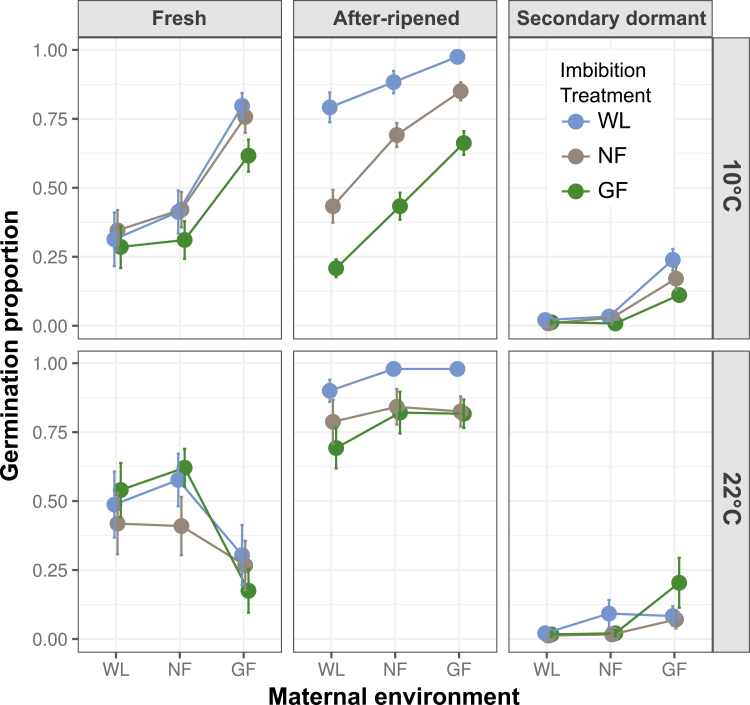
Effect of maturation under white light (WL), a neutral filter (NF) and a green filter (GF) (*x*-axis) on germination of fresh seeds, after-ripened seeds (AR) and seeds induced into secondary dormancy (SD) of the L*er*-*FLC* genotype incubated under WL, NF or GF (see key), and at either 10 °C (upper panel) or 22 °C (lower panel). For statistical significance of pairwise comparisons (between maternal and seed imbibition conditions), see **Supporting Information—Table S2**.

**Figure 3.**
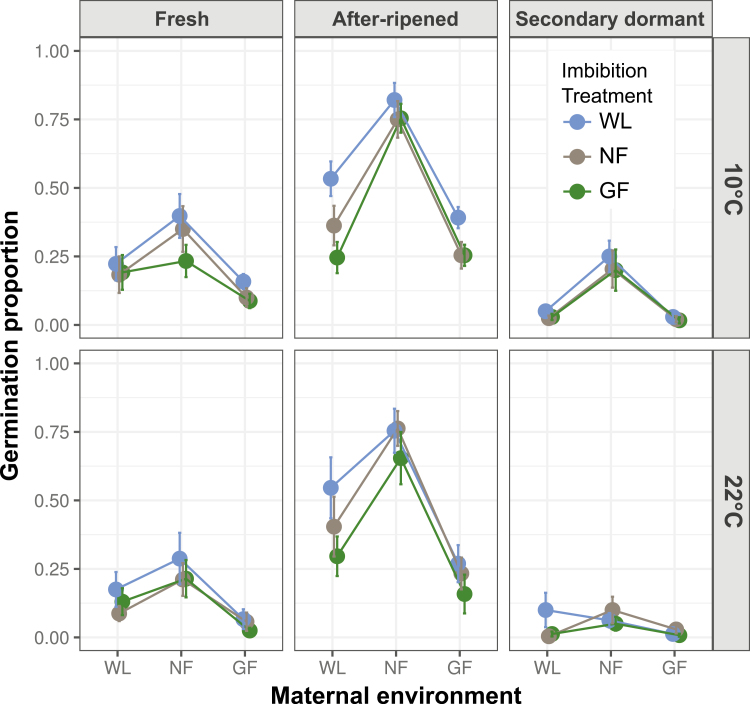
Effect of maturation under white light (WL), a neutral filter (NF) and a green filter (GF) (*x*-axis) on germination of fresh seeds, after-ripened seeds (AR) and seeds induced into secondary dormancy (SD) of the Col genotype imbibed under WL, NF or GF (see key), and at either 10 °C (upper panel) or 22 °C (lower panel). For statistical significance of pairwise comparisons (between maternal and seed imbibition conditions), see **Supporting Information—Table S2**.

Genotypes differed significantly in their germination responses to light experienced during seed maturation and seed imbibition (significant Geno × Mat and Geno × Imbibe interactions; Table 1), although differences with Col could be confounded with batch (see Methods). For all genotypes, imbibition in complete darkness consistently and significantly repressed germination across all temperatures and dormancy levels, confirming that light is required during imbibition for germination to occur **[see****Supporting Information—Fig. S2****]**. Induction of secondary dormancy by hot stratification was effective in all genotypes, strongly reducing germination proportions and variance among treatments (Figs 1–3). Because of such low germination in the dark and in seeds with secondary dormancy, the full model did not converge when those treatments were included, so they were dropped from the analysis (Table 1). To interpret interactions with genotype, we next present results for the genotypes L*er*, L*er-FLC* and Col separately. See supplementary information for the other genotypes **[see****Supporting Information—Supplementary Text**; **Supporting Information—Tables S3 and S4**; **Supporting Information—Figs S6 and S7****]**.

### Germination responses to light conditions: L*er* genotype

In L*er*, maternal light treatments significantly influenced germination, such that germination was enhanced by a combination of reduced irradiance and reduced R:FR during seed maturation (WL vs. GF in Fig. 1; Tables 2 and 3 and **Supporting Information—Table S2a**). The effects of maternal light conditions varied with imbibition temperature within each dormancy treatment (Temp × Mat; Table 2); Mat interacted with after-ripening duration and with secondary-dormancy induction (Mat × AR and Mat × SD in Table 2) in a manner that depended on temperature and imbibition light treatment (Mat × Temp × AR/SD and Mat × Imbibe ×AR/SD in Table 2). A reduction in maternal R:FR (NF vs. GF) was more effective in inducing germination than a reduction in maternal light quantity (WL vs. NF) in almost all imbibition conditions tested (Fig. 1 and **Supporting Information—Fig. S3**; **Supporting Information—Table S2a**). The promotive effect of the simulated canopy appears to be caused primarily by reduced R:FR in seeds with dormancy (Fresh and SD), but a combination of reduced R:FR and reduced irradiance in after-ripened seeds **[see****Supporting Information—Table S2a****]**.

**Table 2.** Results of analyses of the genotypes L*er*, L*er*-*FLC* and Col based on generalized linear models to test for effects of temperature (‘Temp’), maternal light treatment (‘Mat’), seed imbibition light treatment (‘Imbibe’) and their interactions for each dormancy treatment separately (Fresh, AR, SD). The last two columns test for interactions with after-ripening (Fresh vs. AR) and secondary-dormancy induction (AR vs. SD). Analyses are based on logit-linked generalized linear models. Likelihood ratios were tested based on chi-squares. *P < 0.05, **P < 0.01, ***P < 0.001.

	df	Fresh	AR	SD	× AR (Fresh vs. AR)	× SD (AR vs. SD)
		LR chi-sq	LR chi-sq	LR chi-sq	LR chi-sq	LR chi-sq
L*er*						
Temp	1	15.6***	36.1***	23.1***	0.2	7.0**
Mat	2	75.8***	231.1***	64.3***	410.5**	11.2**
Imbibe	2	3.1	130.1***	2.6	46.6***	6.5*
Temp × Mat	2	27.7***	81.8***	1.2	87.1***	14.8***
Temp × Imbibe	2	14.8***	27.9***	1.1	3.5	6.8*
Mat × Imbibe	4	17.1**	21.4***	10.7*	24.7***	10.6*
Temp × Mat × Imbibe	4	16.8**	2.5	20.3***	11.1*	15.3**
L*er*-*FLC*						
Temp	1	15.8***	81.2***	0.1	96.8***	17.4***
Mat	2	20.5***	170.3***	195.4***	63.4***	26.7***
Imbibe	2	14.3***	397.8***	10.5**	185.8***	33.1***
Temp × Mat	2	410.7***	19.6***	7.5*	26.1***	0.9
Temp × Imbibe	2	21.3***	22.4***	3.8	0.5	0.1
Mat × Imbibe	4	26.1***	6.8	11.6*	11.9*	10.8*
Temp × Mat × Imbibe	4	9.4	8.5	9.7*	10.7*	3.7
Col						
Temp	1	49.8***	6.7**	16.0***	16.4***	8.4**
Mat	2	221.3***	782.2***	138.8***	48.4***	1.1
Imbibe	2	26.3***	81.4***	9.6**	1.8	0.3
Temp × Mat	2	5.0	13.8	5.0	4.9	4.4
Temp × Imbibe	2	0.3	5.3	1.0	3.3	1.4
Mat × Imbibe	4	7.9	13.4**	22.3***	16.1**	11.8*
Temp × Mat × Imbibe	4	5.8	3.0	12.9*	4.5	10.0*

**Table 3.** Results of analyses of the genotypes L*er*, L*er*-*FLC* and Col, based on generalized linear models, to test for effects of maternal light treatment (‘Mat’), seed imbibition light treatment (‘Imbibe’), and their interactions, on germination proportion for each combination of temperature and dormancy treatment. Results are based on logit-linked generalized linear models. Likelihood ratios were tested based on chi-squares. Significance levels are expressed as *P < 0.05, **P < 0.01, ***P < 0.001.

	df	Fresh		AR		SD	
		10 °C	22 °C	10 °C	22 °C	10 °C	22 °C
		LR chi-sq	LR chi-sq	LR chi-sq	LR chi-sq	LR chi-sq	LR chi-sq
L*er*							
Mat	2	94.1***	7.0*	21.3***	289.1***	25.6***	52.7***
Imbibe	3	3.7	16.2***	159.1***	18.8***	2.3	1.6
Mat × Imbibe	6	0.9	38.6***	10.0*	13.6**	9.6*	29.7***
L*er*-*FLC*							
Mat	2	288.9***	144.7***	208.8***	34.3***	159.6***	61.0***
Imbibe	3	24.3***	12.6**	357.8***	119.3***	7.5*	7.05*
Mat × Imbibe	6	7.0	27.3***	2.9	10.7*	2.1	27.6***
Col							
Mat	2	94.3***	127.0***	394.1***	399.1***	200.7***	32.0***
Imbibe	3	16.2***	11.8**	46.3***	40.6***	3.9	6.0*
Mat × Imbibe	6	5.9	6.7	10*	6.3	0.9	32.1***

Regarding responses to imbibition light treatments, seeds imbibed under a simulated canopy (WL vs. GF) had decreased germination under some conditions compared to WL (Fig. 1 and **Supporting Information—Fig. S3**; **Supporting Information—Table S2b**). In most cases, this effect appears to be caused primarily by reduced irradiance (WL vs. NF). The reduction in germination was greater at 10 °C than at 22 °C especially in after-ripened seeds.

Responses to maternal light treatments were in general stronger than responses to light treatments during imbibition, and they acted in opposition to each other **[see****Supporting Information—Fig. S3**; **Supporting Information—Table S2****]**. The combination of reduced irradiance plus reduced R:FR during maturation increased germination (with few exceptions), but when those conditions were experienced during imbibition, they decreased germination under certain conditions (Fig. 1 and **Supporting Information—Fig. S3**). Significant interactions between maturation and imbibition treatments in all but fresh seeds incubated at 10 °C (Table 3) indicate that the magnitude responses to imbibition light environment depended on the seed-maturation environment, and vice versa.

### Germination responses to light conditions: L*er*-*FLC* genotype

Maternal light treatments significantly affected germination, but their effects varied with temperature (Temp × Mat; Table 2) and dormancy treatment (significant Mat × AR and Mat × SD; Table 2). The combination of reduced irradiance and reduced R:FR experienced during maturation (WL vs. GF) increased germination in most treatments, as in L*er* (Fig. 2). As before, this effect appears to be caused primarily by reduced R:FR, especially in seeds with dormancy (Fresh and SD), but after-ripened seeds also germinated more when irradiance was reduced (WL vs. NF; **Supporting Information—Table S2a**). A pronounced interaction was observed between maternal treatment and temperature, especially in fresh seeds. Reduced maternal R:FR significantly increased germination of seeds incubated at 10 °C (in all seed treatments), but at 22 °C it decreased germination of fresh seeds (Fig. 2 and **Supporting Information—Fig. S4**; **Supporting Information—Table S2a**).

Regarding responses to imbibition light treatments, seeds imbibed under a simulated canopy (WL vs. GF) had decreased germination under some conditions (especially at 10 °C). The magnitude of the responses to light treatments during imbibition varied with temperature (significant Temp × Imbibe for fresh and after-ripened seeds; Table 2), after-ripening (Imbibe × AR; Table 2) and dormancy induction (Imbibe × SD; Table 2). Reduced irradiance during imbibition decreased germination in after-ripened seeds at both 10 and 22 °C (Fig. 2 and **Supporting Information—Fig. S4**; **Supporting Information—Table S2b**). Reduced R:FR during imbibition (NF vs. GF) sometimes decreased germination in fresh and after-ripened seeds incubated at 10 °C **[see****Supporting Information—Table S2b****]**. At 22 °C, reduced R:FR sometimes increased germination (fresh seeds NF-maternal and SD seeds GF-maternal).

Responses to light treatments experienced in the maternal generation were stronger (and were significant across more treatments) than responses to light treatments experienced during imbibition, and they acted in the opposite direction of each other when seeds were incubated at 10 °C **[see****Supporting Information—Fig. S4**; **Supporting Information—Table S2**]. At this temperature (and for after-ripened seeds at 22 °C), simulated canopy increased germination if experienced during maturation, but it reduced germination when experienced during imbibition (Fig. 2 and **Supporting Information—Fig. S4**). Maternal and imbibition treatments interacted to regulate progeny germination when seeds were incubated at 22 °C (Table 3), in which the response to maternal R:FR was slightly more pronounced when seeds were imbibed under reduced R:FR (Fig. 2; **Supporting Information—Table S2b**).

### Germination responses to light conditions: Col genotype

Maternal light treatments significantly affected germination within all temperature and dormancy treatments (Tables 2 and 3 and **Supporting Information—Table S2a**), such that reduced irradiance during maturation (WL vs. NF) increased germination in most treatments, while reduced R:FR (NF vs. GF) decreased germination (Fig. 3). These effects were more pronounced in after-ripened seeds (Mat × AR; Table 2). The germination response to reduced R:FR in the maternal generation opposed the response to reduced irradiance, leading to a weak or no overall effect of a simulated canopy (WL vs. GF; **Supporting Information—Table S2a**).

A simulated canopy (WL vs. GF) during imbibition slightly and significantly decreased germination under some conditions, especially in after-ripened seeds (Fig. 3), and this effect is attributable to a reduction in irradiance (WL vs. NF), since reduced R:FR (NF vs. GF) had no significant effect **[see****Supporting Information—Table S2b****]**.

Responses to maternal light treatments were stronger (and significant under more conditions) than responses to light treatments during imbibition (Fig. 3 and **Supporting Information—Fig. S5**; Tables 2 and 3 and **Supporting Information—Table S2**). Responses to reduced irradiance in maternal and progeny generations opposed each other; reduced irradiance during maturation increased germination while reduced irradiance during imbibition decreased germination. Responses to reduced R:FR in maternal and progeny generations were in the same direction **[see****Supporting Information—Fig. S5****]**, both reducing germination. Maternal and seed imbibition treatments interacted significantly in after-ripened seeds incubated at 10 °C and seeds induced into secondary dormancy incubated at 22 °C (Table 3), in which responses to imbibition treatments were more pronounced when seeds were matured under white light.

### Effect of dormancy on germination responses

As discussed above, dormancy treatment sometimes influenced the magnitude of responses to maternal and especially imbibition light treatments (Table 2). In general, responses tended to be most pronounced in seeds with the least dormancy (after-ripened), suggesting that dormancy prevents seeds from responding to promotive light conditions.

Regarding genetic manipulations of dormancy/germination, in general, disruption of the germination-promoting (dormancy-impeding) gene, *FLC*, decreased germination on both genetic backgrounds, but the effect was detectable only under very specific light conditions **[see****Supporting Information—Figs S6 and S7**; **Supporting Information—Tables S3** and **S4****]**. *FLC* disruption sometimes altered the response to light experienced during seed maturation by reducing germination under the more permissive light conditions, but it did not significantly alter germination responses to imbibition light conditions **[see****Supporting Information—Table S3****]**. More information is supplied as supplementary material **[see****Supporting Information—Supplementary Text****]**.

## Discussion

Germination responses to a simulated vegetation canopy comprised responses to reductions in both total irradiance and R:FR. Depending on the genetic line, responses to irradiance and R:FR either reinforced or opposed each other. Reduced irradiance and R:FR experienced in the maternal generation had a stronger effect on germination than those experienced during imbibition, and response to maternal light conditions often opposed responses to seed light conditions.

### Germination response to irradiance vs. R:FR

Germination responses to a simulated vegetation canopy were the result of responses to reductions in both total irradiance and R:FR. The distinct responses to each component of the light environment indicate that seeds can distinguish the presence of plant neighbours from that of a neutral shade source and respond to those two cues differently. Our results accord with other studies that have documented distinct responses to reductions in irradiance (light quantity) and reductions in R:FR (light quality) in different species (Fenner 1980; Dobarro *et al.* 2010). Altogether, our results are consistent with distinct responses to different components of the light environment, with phytochrome mediating responses to R:FR, whereas other photoreceptors (likely blue light photoreceptors) appear to mediate responses to total light irradiance (Casal and Sánchez 1998; Smith 2000; Casal 2013).

We observed variation among genotypes in the response to the different components of the light environment. In the L*er* background, responses to reduced irradiance and R:FR reinforced each other to produce a larger response to simulated canopy than either response alone. In contrast, in the Col background, responses to irradiance and R:FR opposed each other, leading to a weak or no net response to simulated canopy. Thus, genotypic differences in responses to vegetation canopy are not necessarily caused by differences in the ability to respond to light conditions in general, but instead can be caused by differences in whether responses to light quantity and light quality complement or antagonize each other. In this study, both genetic backgrounds were able to perceive and respond to both components of light (irradiance and R:FR), but components of the signal transduction pathway (perception or transduction) appear to have diverged. This divergence was apparent specifically in the phytochrome-mediated pathway that increased germination under low R:FR during seed maturation in the L*er* background but decreased germination in Col. Ecotypic differences in responses of other traits to the same light environment have been reported before in *A. thaliana*. Divergence in responses can be attributed to sequence variation in the photoreceptors themselves (El-Din El-Assal *et al.* 2001; Maloof *et al.* 2001; Balasubramanian *et al.* 2006), or to variation in interactions between photoreceptors (Sánchez-Lamas *et al.* 2016). The contribution to variation in progeny responses to maternal environments that may be caused by phytochromes or other photoreceptors, individually or through interactions with each other and/or downstream elements of the signalling pathway, remains to be explored.

It should be noted that some aspects of the experimental execution may compromise our interpretation of responses to irradiance vs. R:FR. First, our filters did not perfectly match R:FR between white light and neutral shade, nor did they perfectly match irradiance levels between the neutral shade and R:FR treatments (see Methods). When the response to reduced R:FR is in the same direction as the response to reduced irradiance, some of the apparent effects of reduced R:FR may be attributed to the small additional reduction in irradiance. However, when reduction of R:FR elicited the opposite response as reduction of irradiance, as it was observed in Col, the response to R:FR cannot be attributed to changes in irradiance. We therefore interpret responses to green vs. neutral filter to reflect responses to large differences in R:FR as opposed to small differences in irradiance. Second, for the L*er* background, the comparison between neutral and green filter could be compromised because the parental plants matured faster under the green filter than under the neutral filter, which confounds the timing of the germination trial with the maternal treatment (although the duration of after-ripening was controlled for). However, our results are consistent with findings from Leverett *et al.* (2016), in that seeds matured under green filter had higher germination than those in white light. The higher germination of seeds matured under green filter observed in this study is likely to be caused by differences in the maternal treatment rather than by differences in the timing of the germination assays that occurred under common conditions.

In terms of adaptive significance, if dormancy is costly for parents to induce in progeny, germination would be expected to be higher when seeds are matured in neutral shade (an environment with resource limitation) than in white light. This outcome was observed in both genetic backgrounds. However, germination was lower when seeds were imbibed under reduced irradiance (NF) than under white light, suggesting either that the maintenance of dormancy in seeds is less costly than its initial induction by maternal parents, or that responses to light quantity do not reflect resource limitation but instead represent a cued response to neutral shade. For example, it is possible that neutral shade created by a fixed object will remain constant over time, such that decreasing germination would not necessarily allow germinants to escape shading; the maternally induced response of germinating sooner rather than later may be advantageous, all else being equal, and provide plants a longer growing period. Responding to R:FR, in contrast, entails responding to potential competition that is likely to increase or decrease over time as the vegetation canopy grows or senesces. Other studies have shown that some seeds are more likely to germinate quickly in the presence of neighbours, including other seeds—seed leachates from con- or heterospecific high density seed clusters may affect germination proportion and timing by establishing an early signal of potential future competition for light as a resource (e.g. Miller *et al.* 1994; Murray 1998; Mercer *et al.* 2011; Weis *et al.* 2015). As such, two strategies are possible: seeds may increase germination or germinate more quickly and thereby outcompete their present or future neighbours via the known ‘shade-avoidance response’, as was observed in the L*er* background. Alternatively, seeds may decrease germination, postponing it until competition is less intense, as was seen in Col. In natural conditions, early autumn germination has been shown to increase the chance of survival to reproduction and fecundity, although it reduces seedling survival (Leverett *et al.* 2018). On the other hand, germinating later increases the facilitative effects of neighbours, which in turn would increase the chances of seedling survival when facing adverse environmental conditions (Leverett *et al.* 2018). It would be interesting to test in additional natural ecotypes whether the direction of the response to R:FR depends on seed germination season, and specifically whether the vegetation canopy is likely to intensify or senesce.

### Within- and trans-generational plasticity

Reduced irradiance and R:FR during seed maturation had a stronger effect on germination response than reduced irradiance and R:FR during seed imbibition. In other words, seeds responded more strongly to light signals from their parent’s environment than to light signals from their own environment. Moreover, responses to progeny environments did not override effects of maternal environment, even when they acted in the opposite direction, as they frequently did. This finding may seem counter-intuitive, since the progeny environment is often thought to be a better predictor of the seedling environment than is the maternal environment (DeWitt *et al.* 1998; Donohue *et al.* 2010; Baskin and Baskin 2014) because of less time for environmental change between environmental perception and natural selection (Ezard *et al.* 2014; English *et al.* 2015). Our results raise the possibility that the maternal environment may actually be a better predictor of future competition for the seedling than the progeny environment at the time of germination (Auge *et al.* 2017b). This is especially the case in cyclical environments (Marshall and Uller 2007; Uller 2008; Ezard *et al.* 2014; Prizak *et al.* 2014; Kuijper and Hoyle 2015), such as seasonal environments when the vegetation canopy might not be present at the seed or seedling stage but may develop later. If the maternal environment experiences reductions in irradiance and R:FR, indicating limited light resources later in the growing season, then responding by stimulating germination may allow the progeny to better compete with its future neighbours for those limited light resources (Cohen 1966; Baskin and Baskin 2014; Leverett *et al.* 2016). Thus, maternal environmental conditions may provide reliable cues of future conditions in cyclically varying environments, such as seasonal environments. Future field studies could experimentally test the conditions under which the maternal or immediate progeny environments are a better predictor of selective environments experienced by progeny.

### Effect of dormancy on germination responses to light

Dormancy influenced responses to light environments. Seeds with primary or secondary dormancy tended to have weaker responses to light, as did genotypes with higher innate (genotypically determined) dormancy. In particular, when genotypes had detectable germination responses, the *FLC* genotypes that impeded a strong dormancy induction/maintenance (active *FLC* genotypes) tended to have more pronounced responses to light. In summary, dormancy appears to inhibit responses to light conditions by preventing germination under promotive light conditions.

The effect of dormancy on responses to light likely has consequences for germination timing under natural conditions. Dormancy is likely to be a general regulator of germination responses to environmental cues, preventing germination under ephemerally promotive conditions, and as physiological dormancy is lost, germination frequently proceeds under an increasingly wide range of environments (Bewley 1997; Baskin and Baskin 2014). If seeds are shed in a dormant state, dormancy can prevent seeds from responding to promotive light cues until they lose that primary dormancy. In *A. thaliana*, which typically flowers in spring and germinates in autumn, this contingency implies that, even if light conditions are favourable for growth in springtime, germination will be impeded then; only in the autumn, after dormancy loss through after-ripening, will seeds be able to respond to promotive light cues for germination. The observation that genetic differences in dormancy also influence germination responses to light suggests that natural genetic variation in the ability to respond to light cues may in part be caused by genetic variation in innate dormancy, affecting in turn how maternal effects are expressed in the next generation.

## Conclusions

Germination responds both to changes in light irradiance and R:FR, and the combination of these responses determines the response to vegetation canopy. Therefore, seeds detect and respond to the presence of neighbours separately from neutral shade. In some genotypes, responses to reduced irradiance and R:FR reinforced each other, while in another they cancelled each other out, suggesting that genetic variation in responses to vegetation canopy need not be caused by differences in the ability to respond or perceive light cues, but rather by differences in the direction and relative magnitude of responses to different components of vegetation shade.

Seeds responded more strongly to light signals experienced by parents than to light signals from their own environment, and frequently in the opposite direction. This result suggests that information acquired at different points in time (during seed maturation vs. during imbibition) has different predictive value regarding the environment of natural selection that progeny will be exposed to. In environments that change within the lifetime of an individual, such as seasonal environments, understanding how cues perceived at different times predict future selective environments will be essential for understanding the adaptive significance of within- and trans-generational plasticity. Under conditions of climate change or range expansion, in which the predictive value of specific cues is likely to change, within and trans-generational plasticity may have important consequences to the ability of populations to colonize or persist in altered seasonal environments.

## Sources of Funding

Our work was funded by NSF-IOS-11-46383 to K.D.

## Contributions by the Authors

G.A.A. and K.D. designed experiments. G.A.A. directed the experiments. K.V. and G.A.A. conducted the experiments. K.V. and G.A.A. analysed the data. K.V., K.D. and G.A.A. interpreted the data. K.V. drafted the manuscript. K.D. and G.A.A. contributed to the final version of the manuscript.

## Conflict of Interest

None declared.

## Supporting Information

The following additional information is available in the online version of this article—

**Table S1**. The eight genotypes used in the study. ‘L*er*’ indicates the Landsberg-*erecta* background; ‘Col’ indicates the Columbia background. Upper- or lower-case *FRI* and *FLC* indicate if the allele is functional or not, respectively.

**Table S2**. Effects of maternal and imbibition light treatments on germination of L*er*, L*er-FLC* and Col seeds. Results of generalized linear models to test for effects of (a) maternal light treatment (‘Mat’) and (b) seed imbibition light treatment (‘Imbibe’) on germination proportion for each combination of temperature and dormancy treatments in the genotypes L*er*, L*er*-*FLC* and Col. Tables show results of pairwise comparisons to test for effects of reduced irradiance (WL vs. NF), presence of a simulated canopy (WL vs. GF) and effect of reduced R:FR (NF vs. GF). In addition, for seed imbibition, WL vs. D tests the overall light requirement during seed incubation (in b). Results are based on logit-linked generalized linear models. Likelihood ratios were tested based on chi-squares. Significance levels are expressed as *P < 0.05, **P < 0.01, ***P < 0.001. For ‘Imbibe’ and ‘Maternal’ columns: D, darkness; WL, white light, control; NF, neutral filter, reduced irradiance; GF, green filter, reduced R:FR. For ‘Dormancy’ column: FS, fresh seeds; AR, 5-months after-ripened seeds; SD, AR seeds induced into a secondary dormant state with hot stratification (see Methods).

**Table S3**. Results of full models for each genetic background (L*er* and Col) to test for effects of manipulation of *FLC*. Full models test for effects of genotype (‘Geno’), temperature (‘Temp’: 10 and 22 °C), maternal light treatment (‘Mat’), seed imbibition light treatment (‘Imbibe’) and after-ripening treatment (‘AR’: Fresh vs. AR), and the effects of their interactions on germination proportions. Germination in darkness and in seeds induced into secondary dormancy were low and had very low variance, so these were removed from the analysis. Results show analyses based on logit-linked generalized linear models. Likelihood ratios were tested based on chi-squares. Reference levels were L*er-FLC* and Col (Geno for L*er* and Col backgrounds, respectively), 10 °C (Temp), NF (Mat), NF (Imbibe) and fresh seeds (AR). Significance levels are expressed as *P < 0.05, **P < 0.01, ***P < 0.001.

**Table S4**. Effects of *FLC* activity on germination in the L*er* and Col backgrounds. Results of generalized linear models on germination proportion for each combination of maternal (‘Mat’), imbibition light treatment (‘Imbibe’), imbibition temperature (‘Temp’) and dormancy (Fresh, After-ripened, Secondary dormant) to test for genotypic effects due to allelic variation in *FLC* of genotypes in (a) L*er* and (b) Col backgrounds. Tables show results for pairwise comparisons to test for effects of non-functional/weak/knocked-down *FLC* alleles compared to functional *FLC* (see Methods, and see **Supporting Information—Table S1** or information on the genotypes). Results are based on logit-linked generalized linear models. Likelihood ratios were tested based on chi-squares. Significance levels are expressed as *P < 0.05, **P < 0.01, ***P < 0.001. For ‘Imbibe’ and ‘Maternal’ columns: D, darkness; WL, white light, control; NF, neutral filter, reduced irradiance; GF, green filter, reduced R:FR.

**Supplementary Text**. Effect of disrupting *FLC*.

**Figure S1**. Experimental design. Diagram of different maturation light, imbibition light, imbibition temperature and after-ripening/secondary-dormancy treatments used in this study. ‘Comparisons’ indicate the contrasts and their interpretation.

**Figure S7**. Effects of *FLC* activity on germination responses to light of genotypes in the Col background. Effect of maturation under white light (WL), a neutral filter (NF) and a green filter (GF) (*x*-axes) on germination of fresh seeds, after-ripened seeds and seeds induced into secondary dormancy (rows) of genotypes in Col background—Col (fri/FLC), Col-*FRI*_*Sf*_ (FRI/FLC), Col-*FRI*_*Sf*_*flc-3* (FRI/flc) and *flc-3* (fri/flc)—imbibed under WL, NF or GF, or kept in darkness (D) (columns), and at either 10 °C (a) or 22 °C (b). See **Supporting Information—Table S1** for information on the genotypes and the **Supporting Information—Supplementary Text** for a discussion of these results.

**Figure S2**. Effect of maturation under white light (WL), a neutral filter (NF) and a green filter (GF) (*x*-axis) on germination of fresh seeds, after-ripened seeds and seeds induced into secondary dormancy of the L*er*, L*er-FLC* and Col genotypes (see key) kept in darkness at either 10 °C (upper panel) or 22 °C (lower panel). For statistical significance of pairwise comparisons (between maternal and seed imbibition conditions), see **Supporting Information—Table S2**.

**Figure S3**. Direction and strength of the effect of seed maturation and imbibition under different light conditions at 10 or 22 °C for the L*er* genotype. Comparisons of WL vs. NF, WL vs. GF, and NF vs. GF were made to assess the strength and direction of the maternal light environment of seeds incubated in WL (‘Maternal light’, grey symbols), and of the imbibition light environment of seeds matured under WL (‘Imbibition light’, black symbols). Rows indicate effects for fresh (upper row) or after-ripened seeds (lower row). Each value is the change in log odds with associated 97.5 % confidence intervals of germination caused by changes in light environment during maturation and imbibition: positive values indicate that each environment (in column order: NF, GF and GF) increases germination compared to the reference environment (in column order: WL, WL and NF). Confidence intervals that cross zero (vertical dashed grey line) indicate there was no effect of the environment.

**Figure S4**. Direction and strength of the effect of seed maturation and imbibition under different light conditions at 10 or 22 °C for the L*er-FLC* genotype. Comparisons of WL vs. NF, WL vs. GF, and NF vs. GF were made to assess the strength and direction of the maternal light environment of seeds incubated in WL (‘Maternal light’, grey symbols), and of the imbibition light environment of seeds matured under WL (‘Imbibition light’, black symbols). Rows indicate effects for fresh (upper row) or after-ripened seeds (lower row). Each value is the change in log odds with associated 97.5 % confidence intervals of germination caused by changes in light environment during maturation and imbibition: positive values indicate that each environment (in column order: NF, GF and GF) increases germination compared to the reference environment (in column order: WL, WL and NF). Confidence intervals that cross zero (vertical dashed grey line) indicate there was no effect of the environment.

**Figure S5**. Direction and strength of the effect of seed maturation and imbibition under different light conditions at 10 or 22 °C for the Col genotype. Comparisons of WL vs. NF, WL vs. GF, and NF vs. GF were made to assess the strength and direction of the maternal light environment of seeds incubated in WL (‘Maternal light’, grey symbols), and of the imbibition light environment of seeds matured under WL (‘Imbibition light’, black symbols). Rows indicate effects for fresh (upper row) or after-ripened seeds (lower row). Each value is the change in log odds with associated 97.5 % confidence intervals of germination caused by changes in light environment during maturation and imbibition: positive values indicate that each environment (in column order: NF, GF and GF) increases germination compared to the reference environment (in column order: WL, WL and NF). Confidence intervals that cross zero (vertical dashed grey line) indicate there was no effect of the environment.

**Figure S6**. Effects of *FLC* activity on germination responses to light of genotypes in the L*er* background. Effect of maturation under white light (WL), a neutral filter (NF) and a green filter (GF) (*x*-axes) on germination of fresh seeds, after-ripened seeds and seeds induced into secondary dormancy (rows) of genotypes in L*er* background—L*er*, L*er-FLC*, L*er-FLC* RNAi-FLC #1 (RNAi #1) and RNAi #2—imbibed under WL, NF or GF, or kept in darkness (D) (columns), and at either 10 °C (a) or 22 °C (b). See **Supporting Information—Table S1** for information on the genotypes and the **Supporting Information—Supplementary Text** for a discussion of these results.

Supplementary Material

## References

[CIT0001] Alonso-Blanco C, El-Assal SE, Coupland G, Koornneef M 1998 Analysis of natural allelic variation at flowering time loci in the Landsberg *erecta* and Cape Verde Islands ecotypes of *Arabidopsis thaliana*. Genetics 149:749–764. 9611189 10.1093/genetics/149.2.749 PMC1460204

[CIT0002] Auge GA, Blair LK, Burghardt LT, Coughlan J, Edwards B, Leverett LD, Donohue K 2015 Secondary dormancy dynamics depends on primary dormancy status in *Arabidopsis thaliana*.Seed Science Research 25:230–246.

[CIT0003] Auge GA, Blair LK, Neville H, Donohue K 2017a Maternal vernalization and vernalization-pathway genes influence progeny seed germination. The New Phytologist 216:388–400. 28328177 10.1111/nph.14520

[CIT0004] Auge GA, Leverett LD, Edwards B, Donohue K 2017b Adjusting phenotypes via within- and across-generational plasticity. The New Phytologist. doi:10.1111/nph.14495. 10.1111/nph.14495 28262950

[CIT0005] Balasubramanian S, Sureshkumar S, Agrawal M, Michael TP, Wessinger C, Maloof JN, Clark R, Warthmann N, Chory J, Weigel D 2006 The *PHYTOCHROME* C photoreceptor gene mediates natural variation in flowering and growth responses of *Arabidopsis thaliana*. Nature Genetics 38:711–715. 16732287 10.1038/ng1818 PMC1592229

[CIT0006] Baskin CC, Baskin JM 2014 Seeds: ecology, biogeography, and evolution of dormancy and germination, 2nd edn. San Diego, CA: Academic Press.

[CIT0007] Bernareggi G, Carbognani M, Mondoni A, Petraglia A 2016 Seed dormancy and germination changes of snowbed species under climate warming: the role of pre- and post-dispersal temperatures. Annals of Botany 118:529–539. 27390354 10.1093/aob/mcw125 PMC4998984

[CIT0008] Bewley JD 1997 Seed germination and dormancy. The Plant Cell 9:1055–1066. 12237375 10.1105/tpc.9.7.1055 PMC156979

[CIT0009] Blair LK, Auge GA, Donohue K 2017 Effect of *FLOWERING LOCUS C* on seed germination depends on dormancy. Functional Plant Biology 44:493–506. 10.1071/FP16368 32480582

[CIT0010] Burghardt LT, Edwards BR, Donohue K 2016 Multiple paths to similar germination behavior in *Arabidopsis thaliana*. The New Phytologist 209:1301–1312. 26452074 10.1111/nph.13685

[CIT0011] Casal JJ 2013 Photoreceptor signaling networks in plant responses to shade. Annual Review of Plant Biology 64:403–427. 10.1146/annurev-arplant-050312-120221 23373700

[CIT0012] Casal JJ, Sánchez RA 1998 Phytochromes and seed germination. Seed Science Research 8:317–329.

[CIT0013] Chen M, MacGregor DR, Dave A, Florance H, Moore K, Paszkiewicz K, Smirnoff N, Graham IA, Penfield S 2014 Maternal temperature history activates *Flowering Locus T* in fruits to control progeny dormancy according to time of year. Proceedings of the National Academy of Sciences 111:18787–18792. 10.1073/pnas.1412274111 PMC4284563 25516986

[CIT0014] Chiang GCK, Barua D, Kramer EM, Amasino RM, Donohue K 2009 Major flowering time gene, *FLOWERING LOCUS C*, regulates seed germination in *Arabidopsis thaliana*. Proceedings of the National Academy of Sciences 106:11661–11666. 10.1073/pnas.0901367106 PMC2710639 19564609

[CIT0015] Clarke JH, Dean C 1994 Mapping *FRI*, a locus controlling flowering time and vernalization response in *Arabidopsis thaliana*. Molecular and General Genetics 242:81–89. 7904045 10.1007/BF00277351

[CIT0067] Cohen D 1966 Optimizing reproduction in a randomly varying environment. Journal of Theoretical Biology 12(1):119–129. 6015423 10.1016/0022-5193(66)90188-3

[CIT0016] Debieu M, Tang C, Stich B, Sikosek T, Effgen S, Josephs E, Schmitt J, Nordborg M, Koornneef M, de Meaux J 2013 Co-variation between seed dormancy, growth rate and flowering time changes with latitude in *Arabidopsis thaliana*. PLoS One 8:e61075. 23717385 10.1371/journal.pone.0061075 PMC3662791

[CIT0017] Dechaine JM, Gardner G, Weinig C 2009 Phytochromes differentially regulate seed germination responses to light quality and temperature cues during seed maturation. Plant, Cell & Environment 32:1297–1309. 10.1111/j.1365-3040.2009.01998.x 19453482

[CIT0018] Deregibus VA, Casal JJ, Jacobo EJ, Gibson D, Kauffman M, Rodriguez AM 1994 Evidence that heavy grazing may promote the germination of *Lolium multiflorum* seeds via phytochrome-mediated perception of high red/far-red ratios. Functional Ecology 8:536–542.#8232;

[CIT0019] Dewitt TJ, Sih A, Wilson DS 1998 Costs and limits of phenotypic plasticity. Trends in Ecology & Evolution 13:77–81. 21238209 10.1016/s0169-5347(97)01274-3

[CIT0020] Dey S, Proulx SR, Teotónio H 2016 Adaptation to temporally fluctuating environments by the evolution of maternal effects. PLos Biology 14:e1002388. 26910440 10.1371/journal.pbio.1002388 PMC4766184

[CIT0021] Dobarro I, Valladares F, Peco B 2010 Light quality and not quantity segregates germination of grazing increasers from decreasers in Mediterranean grasslands. Acta Oecologica 36:74–79.

[CIT0022] Dorn LA, Pyle EH, Schmitt J 2000 Plasticity to light cues and resources in *Arabidopsis thaliana*: testing for adaptive value and costs. Evolution 54:1982–1994. 11209775 10.1111/j.0014-3820.2000.tb01242.x

[CIT0023] Donohue K 2009 Completing the cycle: maternal effects as the missing link in plant life histories. Philosophical Transactions of the Royal Society of London. Series B, Biological Sciences 364:1059–1074. 19324611 10.1098/rstb.2008.0291 PMC2666684

[CIT0024] Donohue K, Dorn L, Griffith C, Kim E, Aguilera A, Polisetty CR, Schmitt J 2005 The evolutionary ecology of seed germination of *Arabidopsis thaliana*: variable natural selection on germination timing. Evolution 59:758–770. 15926687

[CIT0025] Donohue K, Rubio de Casas R, Burghardt LT, Kovach K, Willis CG 2010 Germination, postgermination adaptation, and species ecological ranges. Annual Review of Ecology, Evolution, and Systematics 41:293–319.

[CIT0026] El-Din El-Assal S, Alonso-Blanco C, Peeters AJM, Raz V, Koornneef M 2001 A QTL for flowering time in *Arabidopsis* reveals a novel allele of *CRY2*.Nature Genetics 29:435–440. 11726930 10.1038/ng767

[CIT0068] English S, Pen I, Shea N, Uller T 2015 The information value of non-genetic inheritance in plants and animals. PLoS One 10(1):e0116996. 25603120 10.1371/journal.pone.0116996 PMC4300080

[CIT0027] Ezard THG, Prizak R, Hoyle RB 2014 The fitness costs of adaptation via phenotypic plasticity and maternal effects. Functional Ecology 28:693–701.

[CIT0028] Fenner M 1980 The inhibition of germination of *Bidens Pilosa* seeds by leaf canopy shade in some natural vegetation types. The New Phytologist 84:95–101.

[CIT0029] Fox J, Weisberg S 2010 An R companion to applied regression. Los Angeles, CA: Sage Publications.

[CIT0030] Galloway LF, Etterson JR 2007 Transgenerational plasticity is adaptive in the wild. Science 318:1134–1136. 18006745 10.1126/science.1148766

[CIT0031] Graeber K, Nakabayashi K, Miatton E, Leubner-Metzger G, Soppe WJ 2012 Molecular mechanisms of seed dormancy. Plant, Cell & Environment 35:1769–1786. 10.1111/j.1365-3040.2012.02542.x 22620982

[CIT0032] Groot MP, Kooke R, Knoben N, Vergeer P, Keurentjes JJ, Ouborg NJ, Verhoeven KJ 2016 Effects of multi-generational stress exposure and offspring environment on the expression and persistence of transgenerational effects in *Arabidopsis thaliana*. PLoS One 11:e0151566. 26982489 10.1371/journal.pone.0151566 PMC4794210

[CIT0033] Gutterman Y 2000 Maternal effects on seeds during development. In: Fenner M, ed. Seeds: the ecology of regeneration in plant communities. Wallingford, UK: CAB International, 59–84.

[CIT0034] Herman JJ, Sultan SE 2016 DNA methylation mediates genetic variation for adaptive transgenerational plasticity. Proceedings of the Royal Society B Biological Sciences 283:2016.0988. 10.1098/rspb.2016.0988 PMC5031651 27629032

[CIT0035] Holeski LM, Jander G, Agrawal AA 2012 Transgenerational defense induction and epigenetic inheritance in plants. Trends in Ecology & Evolution 27:618–626. 22940222 10.1016/j.tree.2012.07.011

[CIT0036] Imaizumi T, Auge G, Donohue K 2017 Photoperiod throughout the maternal life cycle, not photoperiod during seed imbibition, influences germination in *Arabidopsis thaliana*. American Journal of Botany 104:516–526. 28411210 10.3732/ajb.1600389

[CIT0037] Kirkpatrick M, Lande R 1989 The evolution of maternal characters. Evolution 43:485–503. 28568400 10.1111/j.1558-5646.1989.tb04247.x

[CIT0038] Koornneef M, Blankestijn-de Vries H, Hanhart CJ, Soppe WJJ, Peeters T 1994 The phenotype of some late-flowering mutants is enhanced by a locus on chromosome 5 that is not effective in the Landsberg *erecta* wild-type. The Plant Journal 6:911–919.

[CIT0039] Kronholm I, Picó FX, Alonso-Blanco C, Goudet J, de Meaux J 2012 Genetic basis of adaptation in *Arabidopsis thaliana*: local adaptation at the seed dormancy QTL DOG1. Evolution 66:2287–2302. 22759302 10.1111/j.1558-5646.2012.01590.x

[CIT0040] Kuijper B, Hoyle RB 2015 When to rely on maternal effects and when on phenotypic plasticity?Evolution 69:950–968. 25809121 10.1111/evo.12635 PMC4975690

[CIT0041] Lacey EP 1996 Parental effects in *Plantago lanceolata* L. I: a growth chamber experiment to examine pre- and postzygotic temperature effects. Evolution 50:865–878. 28568933 10.1111/j.1558-5646.1996.tb03895.x

[CIT0042] Latzel V, Janeček Š, Doležal J, Klimešová J, Bossdorf O 2014 Adaptive transgenerational plasticity in the perennial *Plantago lanceolata*. Oikos 123:41–46.

[CIT0043] Leverett LD, Auge GA, Bali A, Donohue K 2016 Contrasting germination responses to vegetative canopies experienced in pre- vs. post-dispersal environments. Annals of Botany 118:1175–1186. 27551028 10.1093/aob/mcw166 PMC5091727

[CIT0044] Leverett LD, Schieder Iv GF, Donohue K 2018 The fitness benefits of germinating later than neighbors. American Journal of Botany 105:20–30. 29532928 10.1002/ajb2.1004

[CIT0045] Maloof JN, Borevitz JO, Dabi T, Lutes J, Nehring RB, Redfern JL, Trainer GT, Wilson JM, Asami T, Berry CC, Weigel D, Chory J 2001 Natural variation in light sensitivity of *Arabidopsis*. Nature Genetics 29:441–446. 11726931 10.1038/ng777

[CIT0046] Marshall DJ, Uller T 2007 When is a maternal effect adaptive?Oikos 116:1957–1963.

[CIT0047] Mercer KL, Alexander HM, Snow AA 2011 Selection on seedling emergence timing and size in an annual plant, *Helianthus annuus* (common sunflower, Asteraceae). American Journal of Botany 98:975–985. 21653510 10.3732/ajb.1000408

[CIT0048] Miller T, Winn A, Schemske D 1994 The effects of density and spatial distribution on selection for emergence time in *Prunella vularis* (Lamiaceae). American Journal of Botany 81:1–6.

[CIT0049] Montesinos-Navarro A, Picó FX, Tonsor SJ 2012 Clinal variation in seed traits influencing life cycle timing in *Arabidopsis thaliana*. Evolution 66:3417–3431. 23106707 10.1111/j.1558-5646.2012.01689.x

[CIT0050] Moriuchi KS, Friesen ML, Cordeiro MA, Badri M, Vu WT, Main BJ, Aouani ME, Nuzhdin SV, Strauss SY, von Wettberg EJ 2016 Salinity adaptation and the contribution of parental environmental effects in *Medicago truncatula*. PLoS One 11:e0150350. 26943813 10.1371/journal.pone.0150350 PMC4778912

[CIT0051] Murray BR 1998 Density-dependent germination and the role of seed leachate. Australian Journal of Ecology 23:411–418.

[CIT0052] Postma FM, Lundemo S, Ågren J 2016 Seed dormancy cycling and mortality differ between two locally adapted populations of *Arabidopsis thaliana*. Annals of Botany 117:249–256. 26637384 10.1093/aob/mcv171 PMC4724045

[CIT0053] Prizak R, Ezard TH, Hoyle RB 2014 Fitness consequences of maternal and grandmaternal effects. Ecology and Evolution 4:3139–3145. 25247070 10.1002/ece3.1150 PMC4161186

[CIT0069] R Core Team 2017 R: a language and environment for statistical computing Vienna, Austria: R Foundation for Statistical Computing https://www.R-project.org/.

[CIT0054] Ratcliffe D 1976 Germination characteristics and their inter-and intra-population variability in *Arabidopsis*. Arabidopsis Information Service 13:34–45.

[CIT0055] Sánchez-Lamas M, Lorenzo CD, Cerdán PD 2016 Bottom-up assembly of the phytochrome network. PLoS Genetics 12:e1006413. 27820825 10.1371/journal.pgen.1006413 PMC5098793

[CIT0056] Schlichting CD 1986 The evolution of phenotypic plasticity in plants. Annual Review of Ecology and Systematics 17:667–693.

[CIT0057] Schlichting CD, Pigliucci M 1998 Phenotypic evolution: a reaction norm perspective. Sunderland, MA: CAB Direct.

[CIT0058] Schmitt J 1997 Is photomorphogenic shade avoidance adaptive? Perspectives from population biology. Plant, Cell & Environment 20:826–830.

[CIT0059] Singh P, Dave A, Vaistij FE, Worrall D, Holroyd GH, Wells JG, Kaminski F, Graham IA, Roberts MR 2017 Jasmonic acid-dependent regulation of seed dormancy following maternal herbivory in *Arabidopsis*. The New Phytologist 214:1702–1711. 28332706 10.1111/nph.14525

[CIT0060] Smith H 2000 Phytochromes and light signal perception by plants–an emerging synthesis. Nature 407:585–591. 11034200 10.1038/35036500

[CIT0061] Snell-Rood EC 2013 An overview of the evolutionary causes and consequences of behavioural plasticity. Animal Behaviour 85:1004–1011.

[CIT0062] Sultan SE 2000 Phenotypic plasticity for plant development, function and life history. Trends in Plant Science 5:537–542. 11120476 10.1016/s1360-1385(00)01797-0

[CIT0063] Uller T 2008 Developmental plasticity and the evolution of parental effects. Trends in Ecology & Evolution 23:432–438. 18586350 10.1016/j.tree.2008.04.005

[CIT0064] Vu WT, Chang PL, Moriuchi KS, Friesen ML 2015 Genetic variation of transgenerational plasticity of offspring germination in response to salinity stress and the seed transcriptome of *Medicago truncatula*. BMC Evolutionary Biology 15:59. 25884157 10.1186/s12862-015-0322-4 PMC4406021

[CIT0065] Weis AE, Turner KM, Petro B, Austen EJ, Wadgymar SM 2015 Hard and soft selection on phenology through seasonal shifts in the general and social environments: a study on plant emergence time. Evolution 69:1361–1374. 25929822 10.1111/evo.12677

[CIT0066] Zhang R, Gallagher RS, Shea K 2012 Maternal warming affects early life stages of an invasive thistle. Plant Biology (Stuttgart, Germany) 14:783–788. 10.1111/j.1438-8677.2011.00561.x 22404764

