# Supplementary material for: Within- and trans-generational plasticity: seed germination responses to light quantity and quality
Source: AoB Plants. 2018 Apr 10;10(3):ply023. doi: 10.1093/aobpla/ply023 (PMC5951028; doi:10.1093/aobpla/ply023)
Supplement: Supplementary Material [file ply023_suppl_supplementary-material.docx]

**Supplemental Text: Effect of disrupting *FLC***

Previous results indicate that *FLC* promotes seed germination (Chiang et al. 2009, Blair *et al*. 2017). On the L*er* background, the genotype L*er*-*FLC* contains an introgressed portion of the Cvi accession that includes a highly active allele of *FLC*. L*er*-*FLC* had higher germination than the L*er* wild type and more pronounced responses to light (Fig. S6, Table S4a). Direct disruption of *FLC* via RNAi did reduce germination in some conditions, as had been documented previously (Blair *et al*. 2017), but it did so in only a few treatments, suggesting either that the RNAi manipulation was not effective in some treatments or that the introgressed region of the Cvi chromosome contains genes other than *FLC* that influence germination and its response to light. Differences between the RNAi lines and L*er-FLC* were observed when one or both generations experienced a reduction in light quantity or quality (see Table S4a). Because *FLC* disruption decreased germination, it sometimes reduced the response to light conditions by preventing germination under more permissive conditions (e.g. GF maturation), but it also sometimes enhanced the response to light by reducing germination under the least permissive conditions (WL maturation).

In the Col background, *FLC* disruption decreased germination in general, as has been documented previously (Blair *et al*. 2017, Auge *et al*. 2017b), but this effect was apparent only under a few conditions. On a functional *FRI* background, in which *FLC* expression is predicted to be high, *FLC* disruption (Col-*FRI*_Sf_ vs Col-*FRI*_Sf_ *flc-3*) decreased germination primarily in fresh seeds matured or imbibed under a green filter at both incubation temperatures. On a non-functional *FRI* background, *FLC* disruption (Col vs *flc-3*) decreased germination primarily in after-ripened seeds matured under a neutral filter, either induced or not into secondary dormancy. In general, when significant effects of genotype were observed, disruption of *FLC* weakened responses to maturation light conditions, although even control lines exhibited weak responses to light in some cases; it did not alter responses to imbibition light conditions (Tables S3 and S4b).

Disruption of *FRI* on a functional *FLC* background (Col-*FRI*_Sf_ vs Col) slightly decreased germination of fresh seeds (and after-ripened seeds in some conditions) matured under a green filter and incubated at 22°C, but it increased germination of after-ripened and secondarily dormant seeds matured under a neutral filter or control (WL) conditions (Fig. S7, Table S4b). On a non-functional *FLC* background, disruption of *FRI*  (Col-*FRI*_Sf_ *flc-3* vs *flc-3*) increased germination in some combinations of treatments, mostly when seeds were incubated in darkness. This subtle enhancing effect of *FRI* disruption when *FLC* is non-functional has been observed previously (Blair *et al*. 2017, Auge *et al*. 2017b). Disruption of *FRI* decreased germination in a few instances primarily when seeds were matured or incubated under a neutral filter.

In general, disruption of *FLC* did decrease germination in both genetic backgrounds, consistent with previous results, but the effect was detectable only under very specific light conditions. *FLC* disruption, moreover, sometimes altered the strength of the response to light, either by reducing germination under the more permissive light conditions, thereby reducing the response to light, or by reducing germination under the least permissive conditions, thereby enhancing the response to light.

**Table S1.** The eight genotypes used in the study. “L*er*” indicates the Landsberg-*erecta* background; “Col” indicates the Columbia background. Upper- or lower-case *FRI* and *FLC* indicate if the allele is functional or not, respectively.

| **Genotype** | **Details** | ***FRI*** | ***FLC*** |
| --- | --- | --- | --- |
| *Ler* | Wild type | fri | flc |
| L*er*-*FLC* | Introgressed *FLC* locus | fri | FLC |
| RNAi #1 | Introgressed *FLC* locus + knockout | fri | flc |
| RNAi #2 | Introgressed *FLC* locus + knockout | fri | flc |
| Col | Wild type | fri | FLC |
| Col-*flc*-3 | Knockout | fri | flc |
| Col-*FRI*_Sf_ | Introgressed from San Feliu-2 ecotype | FRI | FLC |
| Col-*FRI*_Sf_-*flc*-3 | Introgressed from San Feliu-2 ecotype + knockout | FRI | flc |

**Table S2.** Effects of maternal and imbibition light treatments on germination of L*er*, L*er-FLC* and Col seeds. Results of generalized linear models to test for effects of **a)** maternal light treatment (“Mat”) and **b)** seed imbibition light treatment (“Imbibe”) on germination proportion for each combination of temperature and dormancy treatments in the genotypes *Ler*, L*er*-*FLC* and Col. Tables show results of pairwise comparisons to test for effects of reduced irradiance (WL vs NF), presence of a simulated canopy (WL vs GF), and effect of reduced R:FR (NF vs GF). In addition, for seed imbibition, WL vs D tests the overall light requirement during seed incubation (in **b**). Results are based on logit-linked generalized linear models. Likelihood ratios were tested based on Chi-squares. Significance levels are expressed as **P*<0.05, ***P*<0.01, ****P*<0.001. For “Imbibe” and “Maternal” columns: D, darkness; WL, white light, control; NF, neutral filter, reduced irradiance; GF, green filter, reduced R:FR. For ‘Dormancy’ column: FS, fresh seeds; AR, 5 months after-ripened seeds; SD, AR seeds induced into a secondary dormant state with hot stratification (see Methods).

**a)**

|  |  | **Mat →** | **WL vs NF** | | | | **WL vs GF** | | | | **NF vs GF** | | | |
| --- | --- | --- | --- | --- | --- | --- | --- | --- | --- | --- | --- | --- | --- | --- |
| **Genotype** | **Imbibe** | **Dormancy** | **10°C** | | **22°C** | | **10°C** | | **22°C** | | **10°C** | | **22°C** | |
| L*er* | D | Fresh | 1.39 |  | 0 |  | 2.78 |  | 0 |  | 0.34 |  | 0 |  |
|  |  | AR | 1.94 |  | 0 |  | 1.05 |  | 0 |  | 0.14 |  | 0 |  |
|  |  | SD | 0 |  | 1.39 |  | 0 |  | 1.39 |  | 0 |  | 0 |  |
|  | WL | Fresh | 0.61 |  | 7.58 |  | 24.96 | *** | 1.04 |  | 18.03 | *** | 14 | ** |
|  |  | AR | 9.08 | * | 34.81 | *** | 1.08 |  | 63.62 | *** | 3.92 |  | 4.56 |  |
|  |  | SD | 2.94 |  | 12.28 | ** | 30.26 | *** | 10.28 | ** | 16.56 | *** | 0.1 |  |
|  | NF | Fresh | 1.45 |  | 7.15 |  | 30.51 | *** | 0.54 |  | 19.36 | *** | 11.54 | ** |
|  |  | AR | 3.93 |  | 14.15 | ** | 13.12 | ** | 93.25 | *** | 2.95 |  | 36.18 | *** |
|  |  | SD | 0.85 |  | 6.1 |  | 2.73 |  | 7.98 | * | 0.55 |  | 26.56 | *** |
|  | GF | Fresh | 0 |  | 0.49 |  | 19.97 | *** | 16.06 | *** | 19.97 | *** | 11.06 | ** |
|  |  | AR | 0.96 |  | 27.55 | *** | 8.72 | * | 139.28 | *** | 3.9 |  | 45.86 | *** |
|  |  | SD | 0.2 |  | 0.34 |  | 2.41 |  | 21.02 | *** | 3.93 |  | 26.1 | *** |
| L*er-FLC* | D | Fresh | 0.69 |  | 12.65 | ** | 26.12 | *** | 0 |  | 19.13 | *** | 12.65 | ** |
|  |  | AR | 0.2 |  | 1.39 |  | 4.99 |  | 0 |  | 7.03 |  | 1.39 |  |
|  |  | SD | 0 |  | 1.39 |  | 1.39 |  | 0 |  | 1.39 |  | 1.39 |  |
|  | WL | Fresh | 4.91 |  | 3.81 |  | 118.3 | *** | 16.98 | *** | 76.72 | *** | 36.73 | *** |
|  |  | AR | 7.49 |  | 14.34 | ** | 44.07 | *** | 14.34 | ** | 16.53 | *** | ~0 |  |
|  |  | SD | 0.72 |  | 12.74 | ** | 57.94 | *** | 10.13 | * | 47.67 | *** | 0.12 |  |
|  | NF | Fresh | 2.86 |  | 0.05 |  | 84.61 | *** | 12.48 | ** | 57.32 | *** | 10.98 | ** |
|  |  | AR | 32.94 | *** | 2.34 |  | 95.02 | *** | 1.08 |  | 17.31 | *** | 0.24 |  |
|  |  | SD | 2.97 |  | 0.14 |  | 46.92 | *** | 11.24 | ** | 29.49 | *** | 9.03 | * |
|  | GF | Fresh | 0.31 |  | 3.13 |  | 54.06 | *** | 72.88 | *** | 46.17 | *** | 104.13 | *** |
|  |  | AR | 28.33 | *** | 11.27 | ** | 104.86 | *** | 10.48 | * | 25.68 | *** | 0.01 |  |
|  |  | SD | 0.2 |  | 0.11 |  | 23.62 | *** | 49.64 | *** | 27.34 | *** | 46.07 | *** |
| Col | D | Fresh | 0.42 |  | 12.82 | ** | 20.49 | *** | 21.35 | *** | 26.02 | *** | 2.61 |  |
|  |  | AR | 11.98 | ** | 2.78 |  | 10.15 | * | 11.22 | * | 0.08 |  | 3.93 |  |
|  |  | SD | 0 |  | 0 |  | 0 |  | 0 |  | 0 |  | 0 |  |
|  | WL | Fresh | 16.28 | ** | 8.46 |  | 3.46 |  | 13.68 | ** | 33.93 | *** | 42.46 | *** |
|  |  | AR | 46.58 | *** | 23.16 | *** | 9.72 | * | 40.23 | *** | 96.56 | *** | 122.54 | *** |
|  |  | SD | 40.59 | *** | 2.32 |  | 1.38 |  | 19.75 | *** | 54.84 | *** | 9.09 | * |
|  | NF | Fresh | 17.27 | *** | 16.98 | *** | 6.94 |  | 1.91 |  | 45.02 | *** | 30.76 | *** |
|  |  | AR | 75.09 | *** | 65.05 | *** | 6.63 |  | 16.28 | ** | 123.39 | *** | 141.51 | *** |
|  |  | SD | 44.04 | *** | 27.42 | *** | 0.09 |  | 5.14 |  | 47.31 | *** | 10.5 | * |
|  | GF | Fresh | 1.24 |  | 6.75 |  | 11.07 | * | 24.06 | *** | 19.55 | *** | 53.72 | *** |
|  |  | AR | 130.02 | *** | 65.64 | *** | 0.04 |  | 13.63 | ** | 125.59 | *** | 129.22 | *** |
|  |  | SD | 38.28 | *** | 5.75 |  | 0.86 |  | 0.65 |  | 48.61 | *** | 7.91 | * |

**b)**

|  |  | **Imbibe →** | **WL vs NF** | | | | **WL vs GF** | | | | **NF vs GF** | | | | **WL vs D** | | | |
| --- | --- | --- | --- | --- | --- | --- | --- | --- | --- | --- | --- | --- | --- | --- | --- | --- | --- | --- |
| **Genotype** | **Maternal** | **Dormancy** | **10°C** | | **22°C** | | **10°C** | | **22°C** | | **10°C** | | **22°C** | | **10°C** | | **22°C** | |
| L*er* | WL | Fresh | 0.19 |  | 0.18 |  | 0.45 |  | 0.02 |  | 0.05 |  | 0.08 |  | 16.94 | ** | 34.53 | *** |
|  |  | AR | 35.8 | *** | 0.43 |  | 46.89 | *** | 13.72 | ** | 0.78 |  | 9.33 | * | 254.6 | *** | 161.28 | *** |
|  |  | SD | 1.95 |  | 3.43 |  | 1.05 |  | 0.08 |  | 0.14 |  | 2.48 |  | 1.39 |  | 4.01 |  |
|  | NF | Fresh | ~0 |  | 32.9 | *** | 2.09 |  | 12.83 | ** | 2.06 |  | 5.08 |  | 16.44 | *** | 12.65 | ** |
|  |  | AR | 51.33 | *** | 7.93 |  | 77.76 | *** | 18.99 | *** | 3.55 |  | 2.41 |  | 316.05 | *** | 311.11 | *** |
|  |  | SD | 0.34 |  | 16.73 | *** | 1.35 |  | 14.34 | ** | 2.99 |  | 0.11 |  | 6.98 |  | 34.53 | *** |
|  | GF | Fresh | 0.05 |  | 1.77 |  | 1.32 |  | 9.91 | * | 1.79 |  | 19.85 | *** | 56.08 | *** | 45.26 | *** |
|  |  | AR | 11.71 | ** | 1.23 |  | 24.65 | *** | 0.1 |  | 2.42 |  | 0.61 |  | 266.63 | *** | 374.96 | *** |
|  |  | SD | 7.94 |  | 2.1 |  | 10.76 | ** | 2.93 |  | 0.23 |  | 0.07 |  | 37.53 | *** | 31.55 | *** |
| L*er-FLC* | WL | Fresh | 0.5 |  | 2.27 |  | 0.48 |  | 1.4 |  | 1.97 |  | 7.25 |  | 36.75 | *** | 200.59 | *** |
|  |  | AR | 66.84 | *** | 11.74 |  | 174.15 | *** | 34.29 | *** | 28.33 | *** | 5.89 |  | 369 | *** | 504.57 | *** |
|  |  | SD | 1.35 |  | 0.51 |  | 0.51 |  | 0.11 |  | 0.2 |  | 0.14 |  | 6.98 |  | 6.98 |  |
|  | NF | Fresh | 0.03 |  | 13.6 | ** | 5.51 |  | 1 |  | 6.41 |  | 21.79 | *** | 179.96 | *** | 188.9 | *** |
|  |  | AR | 27.12 | *** | 31.19 | *** | 115.11 | *** | 37.81 | *** | 32.94 | *** | 0.37 |  | 463.73 | *** | 603.72 | *** |
|  |  | SD | 0.07 |  | 15.05 | ** | 3.93 |  | 12.82 | ** | 2.97 |  | 0.11 |  | 11.22 | * | 25.48 | *** |
|  | GF | Fresh | 1.07 |  | 0.83 |  | 19.03 | *** | 11.09 | * | 11.09 | * | 5.89 |  | 391.57 | *** | 114.36 | *** |
|  |  | AR | 25.83 | *** | 36.47 | *** | 91.34 | *** | 39.17 | *** | 23.35 | *** | 0.06 |  | 519.79 | *** | 616.9 | *** |
|  |  | SD | 3.38 |  | 0.26 |  | 14.37 | ** | 14.48 | ** | 3.71 |  | 18.5 | *** | 78.04 | *** | 28.59 | *** |
| Col | WL | Fresh | 1.28 |  | 8.52 |  | 0.81 |  | 1.88 |  | 0.05 |  | 2.68 |  | 20.5 | *** | 13.68 | ** |
|  |  | AR | 14.23 | ** | 9.69 |  | 42.47 | *** | 32.34 | *** | 7.75 |  | 6.42 |  | 165.67 | *** | 232 | *** |
|  |  | SD | 2.14 |  | 27.42 | *** | 1.38 |  | 19.57 | *** | 0.08 |  | 1.05 |  | 22.36 | *** | 34.53 | *** |
|  | NF | Fresh | 1.14 |  | 3.77 |  | 14.63 | ** | 3.6 |  | 7.95 |  | 0.001 |  | 60.18 | *** | 90.87 | *** |
|  |  | AR | 3.58 |  | 0.04 |  | 3.2 |  | 5.78 |  | 0.01 |  | 6.85 |  | 261.21 | *** | 347.25 | *** |
|  |  | SD | 1.51 |  | 2.32 |  | 1.72 |  | 0.32 |  | 0.01 |  | 4.19 |  | 120.18 | *** | 21.21 | *** |
|  | GF | Fresh | 3.66 |  | 0.2 |  | 5.65 |  | 5.38 |  | 0.22 |  | 3.77 |  | 47.87 | *** | 21.35 | *** |
|  |  | AR | 10.44 | * | 0.85 |  | 10.44 | * | 9.19 |  | ~0 |  | 4.31 |  | 51.85 | *** | 59.93 | *** |
|  |  | SD | 0.34 |  | 1.71 |  | 0.86 |  | 0.2 |  | 0.11 |  | 3.01 |  | 12.95 | ** | 4.15 |  |

**Table S3.** Results of full models for each genetic background (L*er* and Col) to test for effects of manipulation of *FLC*. Full models test for effects of genotype (“Geno”), temperature (“Temp”: 10°C and 22°C), maternal light treatment (“Mat”), seed imbibition light treatment (“Imbibe”) and after-ripening treatment (“AR”: Fresh vs AR), and the effects of their interactions on germination proportions. Germination in darkness and in seeds induced into secondary dormancy were low and had very low variance, so these were removed from the analysis. Results show analyses based on logit-linked generalized linear models. Likelihood ratios were tested based on Chi-squares. Reference levels were L*er-FLC* and Col (Geno for L*er* and Col backgrounds, respectively), 10°C (Temp), NF (Mat), NF (Imbibe) and fresh seeds (AR). Significance levels are expressed as **P*<0.05, ***P*<0.01, ****P*<0.001.

|  |  | **L*er*** | | **Col** | |
| --- | --- | --- | --- | --- | --- |
| **Source of variation** | **df** | **LR Chisq** | | **LR Chisq** | |
| Geno | 3 | 37.2 | *** | 31.1 | *** |
| Temp | 1 | 0.1 |  | 12.2 | *** |
| Mat | 2 | 95.5 | *** | 46.7 | *** |
| Imbibe | 2 | 7.9 | * | 15.6 | *** |
| AR | 1 | 36.1 | *** | 79.9 | *** |
| Geno × Temp | 3 | 34.1 | *** | 19.6 | *** |
| Geno × Mat | 6 | 164.6 | *** | 48.9 | *** |
| Temp × Mat | 2 | 93.1 | *** | 0.4 |  |
| Geno × Imbibe | 6 | 9.5 |  | 8.2 |  |
| Temp × Imbibe | 2 | 25.7 | *** | 4.1 |  |
| Mat × Imbibe | 4 | 7.0 |  | 5.9 |  |
| Geno × AR | 3 | 53.4 | *** | 23.9 | *** |
| Temp × AR | 1 | 9.9 | ** | 6.8 | ** |
| Mat × AR | 2 | 8.3 | * | 7.8 | * |
| Imbibe × AR | 2 | 39.3 | *** | 4.2 |  |
| Geno × Temp × Mat | 6 | 186.2 | *** | 15.9 | * |
| Geno × Temp × Imbibe | 6 | 79.0 | *** | 18.1 | ** |
| Geno × Mat × Imbibe | 12 | 38.2 | *** | 7.5 |  |
| Temp × Mat × Imbibe | 4 | 9.4 |  | 5.8 |  |
| Geno × Temp × AR | 3 | 16.8 | *** | 30.6 | *** |
| Geno × Mat × AR | 6 | 67.4 | *** | 25.2 | *** |
| Temp × Mat × AR | 2 | 5.9 |  | 1.1 |  |
| Geno × Imbibe × AR | 6 | 2.2 |  | 18.8 | ** |
| Temp × Imbibe × AR | 2 | 1.7 |  | 7.6 | * |
| Mat × Imbibe × AR | 4 | 3.3 |  | 13.7 | ** |
| Geno × Temp × Mat × Imbibe | 12 | 60.8 | *** | 24.5 | * |
| Geno × Temp × Mat × AR | 6 | 41.9 | *** | 14.9 | * |
| Geno × Temp × Imbibe × AR | 6 | 12.5 |  | 21.8 | ** |
| Geno × Mat × Imbibe × AR | 12 | 22.7 | * | 18.1 |  |
| Temp × Mat × Imbibe × AR | 4 | 10.7 | * | 4.5 |  |
| Geno × Temp × Mat × Imbibe × AR | 12 | 23.2 | * | 30.6 | ** |

**Table S4.** Effects of *FLC* activity on germination in the L*er* and Col backgrounds. Results of generalized linear models on germination proportion for each combination of maternal (“Mat”), imbibition light treatment (“Imbibe”), imbibition temperature (“Temp”) and dormancy (Fresh, After-ripened, Secondary dormant) to test for genotypic effects due to allelic variation in *FLC* of genotypes in **a)** L*er* and **b)** Col backgrounds. Tables show results for pairwise comparisons to test for effects of non-functional/weak/knocked-down *FLC* alleles compared to functional *FLC* (see Methods and Table S1 for information on the genotypes). Results are based on logit-linked generalized linear models. Likelihood ratios were tested based on Chi-squares. Significance levels are expressed as **P*<0.05, ***P*<0.01, ****P*<0.001. For ‘Imbibe’ and ‘Maternal’ columns: D, darkness; WL, white light, control; NF, neutral filter, reduced irradiance; GF, green filter, reduced R:FR.

**a)**

|  |  |  |  | **Fresh** | | | | **After-ripened** | | | | **Secondary dormant** | | | |
| --- | --- | --- | --- | --- | --- | --- | --- | --- | --- | --- | --- | --- | --- | --- | --- |
| **Geno** | **Ref Geno** | **Mat** | **Imbibe** | **10°C** | | **22°C** | | **10°C** | | **22°C** | | **10°C** | | **22°C** | |
| L*er* | L*er-FLC* | WL | D | 33.14 | *** | 0 |  | 1.05 |  | 0 |  | 0 |  | 1.39 |  |
|  |  |  | WL | 62.03 | *** | 92.65 | *** | 20.26 | *** | 138.4 | *** | 2.94 |  | 0.09 |  |
|  |  |  | NF | 79.29 | *** | 61.14 | *** | 5.08 |  | 85.19 | *** | 0.69 |  | 7.98 |  |
|  |  |  | GF | 61.01 | *** | 112.71 | *** | 4.89 |  | 101.46 | *** | ~0 |  | 0.85 |  |
|  |  | NF | D | 19.13 | ** | 12.65 | * | 0.69 |  | 1.39 |  | 0 |  | 1.39 |  |
|  |  |  | WL | 85.69 | *** | 188.9 | *** | 17.81 | ** | 90.77 | *** | 0.72 |  | 0.08 |  |
|  |  |  | NF | 89.33 | *** | 25.25 | *** | 37.99 | *** | 49.76 | *** | ~0 |  | ~0 |  |
|  |  |  | GF | 69.13 | *** | 135.33 | *** | 4.66 |  | 63.59 | *** | ~0 |  | ~0 |  |
|  |  | GF | D | 0.34 |  | 0 |  | 4.99 |  | 0 |  | 1.39 |  | 0 |  |
|  |  |  | WL | 195.99 | *** | 21.94 | *** | 96.38 | *** | 57.67 | *** | 14.45 | ** | 0.1 |  |
|  |  |  | NF | 158.04 | *** | 25.9 | *** | 71.21 | *** | 0.34 |  | 22.46 | *** | 5.19 |  |
|  |  |  | GF | 114.76 | *** | 3.16 |  | 27.56 | *** | 1.28 |  | 11.78 | * | 3.22 |  |
| RNAi #1 | L*er-FLC* | WL | D | 33.15 | *** | 0 |  | 1.04 |  | 0 |  | 0 |  | 0 |  |
|  |  |  | WL | 2.88 |  | 2.14 |  | 5.94 |  | 6.38 |  | 1.19 |  | 2.38 |  |
|  |  |  | NF | 0.75 |  | 11.12 |  | 2.42 |  | 0.95 |  | 0.34 |  | 29.93 | *** |
|  |  |  | GF | 2.99 |  | 9.94 |  | 8.97 |  | 3.51 |  | ~0 |  | 0.85 |  |
|  |  | NF | D | 12.38 | * | 12.65 | * | ~0 |  | 1.39 |  | 0 |  | 1.39 |  |
|  |  |  | WL | 6.64 |  | 6.48 |  | 1.92 |  | 0.72 |  | 1.39 |  | 7.6 |  |
|  |  |  | NF | 21.3 | *** | 4.99 |  | 1.74 |  | 3.11 |  | 0.33 |  | 5.19 |  |
|  |  |  | GF | 2.11 |  | 123.7 | *** | 17.03 | ** | 12.29 | * | 2.99 |  | 0.73 |  |
|  |  | GF | D | ~0 |  | 0 |  | 0.93 |  | 1.37 |  | 1.39 |  | 0 |  |
|  |  |  | WL | 0.06 |  | 21.94 | *** | 10.29 |  | 17.7 | ** | 6.68 |  | 2.83 |  |
|  |  |  | NF | 4.81 |  | 25.9 | *** | ~0 |  | 2.52 |  | 0.38 |  | 4.51 |  |
|  |  |  | GF | 8.21 |  | 3.16 |  | 0.45 |  | 9.58 |  | 3.07 |  | 10.15 |  |
| RNAi #2 | L*er-FLC* | WL | D | 33.15 | *** | 0 |  | 1.04 |  | 0 |  | 0 |  | 0 |  |
|  |  |  | WL | 0.1 |  | 3.73 |  | 10.16 |  | 2.89 |  | 0.72 |  | 0.51 |  |
|  |  |  | NF | 0.26 |  | 14.76 | * | 2.42 |  | 12.62 | * | 0.2 |  | 1.05 |  |
|  |  |  | GF | 23.28 | *** | 121.53 | *** | 3.65 |  | 2.83 |  | ~0 |  | 2.03 |  |
|  |  | NF | D | 25.65 | *** | 12.65 | * | 5.57 |  | 1.39 |  | 0 |  | 1.39 |  |
|  |  |  | WL | 4.64 |  | 0.15 |  | 0.19 |  | 9.14 |  | 2.86 |  | 2.69 |  |
|  |  |  | NF | 0.84 |  | 37.78 | *** | 1.48 |  | 2.34 |  | 6.96 |  | 3.68 |  |
|  |  |  | GF | 3.54 |  | 0.7 |  | 11.46 |  | 35.16 | *** | 2.13 |  | 10.2 |  |
|  |  | GF | D | 1.38 |  | 0 |  | 0.49 |  | 1.39 |  | 1.39 |  | 0 |  |
|  |  |  | WL | 3.22 |  | 3.88 |  | 1.55 |  | 0.11 |  | 6.03 |  | 0.48 |  |
|  |  |  | NF | 3.02 |  | 6.49 |  | 0.02 |  | 6.5 |  | 3.38 |  | 14.04 | * |
|  |  |  | GF | 0.86 |  | 0.34 |  | 0.91 |  | 0.01 |  | 3.07 |  | 25.2 | *** |

**b)**

|  |  |  |  | **Fresh** | | | | **After-ripened** | | | | **Secondary dormant** | | | |
| --- | --- | --- | --- | --- | --- | --- | --- | --- | --- | --- | --- | --- | --- | --- | --- |
| **Geno** | **Ref Geno** | **Mat** | **Imbibe** | **10°C** | | **22°C** | | **10°C** | | **22°C** | | **10°C** | | **22°C** | |
| *FRI* disruption on functional *FLC* (FRI/FLC vs fri/FLC) | | | | | | | | | | | | | | | |
| Col-*FRI*_Sf_ | Col | WL | D | 1.92 |  | 1.03 |  | 4.92 |  | 1.39 |  | 0 |  | 0 |  |
|  |  |  | WL | 1.12 |  | 0.01 |  | 0.07 |  | 43.3 | *** | 4.36 |  | 14.34 | ** |
|  |  |  | NF | 0.01 |  | 9.14 |  | 0.99 |  | 31.52 | *** | 0.4 |  | 5.11 |  |
|  |  |  | GF | 0.01 |  | 0.06 |  | 0.04 |  | 0.63 |  | 0.08 |  | ~0 |  |
|  |  | NF | D | 8.07 |  | ~0 |  | 3.5 |  | 0 |  | 1.39 |  | 1.39 |  |
|  |  |  | WL | 5.34 |  | 4.89 |  | 7.93 |  | 20.29 | *** | 19.22 | *** | 0.31 |  |
|  |  |  | NF | 8.56 |  | 5.08 |  | 19.68 | ** | 65.06 | *** | 8.73 |  | 4.4 |  |
|  |  |  | GF | 0.19 |  | 0.16 |  | 63.22 | *** | 8.3 |  | 8.67 |  | 0.18 |  |
|  |  | GF | D | 17.78 | ** | 11.87 |  | 6.43 |  | 11.22 |  | 0 |  | 0 |  |
|  |  |  | WL | 6.66 |  | 25.31 | *** | 16.99 | ** | 15.31 | * | 0.07 |  | 0 |  |
|  |  |  | NF | 5.75 |  | 13.42 | * | 6.63 |  | 0.01 |  | 0.09 |  | 0.55 |  |
|  |  |  | GF | 5.49 |  | 19.65 | ** | 0.4 |  | 6.24 |  | ~0 |  | 11.61 | * |
| *FLC* disruption on non-functional *FRI* (fri/FLC vs fri/flc) | | | | | | | | | | | | | | | |
| Col | *flc-3* | WL | D | 13.53 | * | 16.44 | ** | ~0 |  | 0 |  | 0 |  | 0 |  |
|  |  |  | WL | 2.08 |  | 0.22 |  | 2.17 |  | 0.83 |  | 0.59 |  | 19.57 | *** |
|  |  |  | NF | 0.44 |  | 4.47 |  | 0.08 |  | 3.05 |  | 7.53 |  | 2.94 |  |
|  |  |  | GF | 0.26 |  | 4.24 |  | 6.72 |  | 0.16 |  | 0.85 |  | 0.2 |  |
|  |  | NF | D | 2.39 |  | 2.99 |  | 3.22 |  | 0.2 |  | 0 |  | 0 |  |
|  |  |  | WL | 2.4 |  | 7.92 |  | 3.99 |  | 26.76 | *** | 38.24 | *** | 3.08 |  |
|  |  |  | NF | 1.15 |  | 2.16 |  | 25.7 | *** | 33.99 | *** | 9.63 |  | 6.49 |  |
|  |  |  | GF | 0.02 |  | 0.71 |  | 36.85 | *** | 16.49 | ** | 17.72 | ** | 0.32 |  |
|  |  | GF | D | ~0 |  | 0 |  | 5.45 |  | 3.85 |  | 0 |  | 0 |  |
|  |  |  | WL | 7.31 |  | 0.32 |  | 0.01 |  | 0.36 |  | 2.73 |  | 8.07 |  |
|  |  |  | NF | 1.38 |  | ~0 |  | 0.2 |  | 0.44 |  | 1.32 |  | 0.03 |  |
|  |  |  | GF | 5.47 |  | 3.12 |  | 3.53 |  | 0.24 |  | 0.45 |  | ~0 |  |
| *FLC* disruption on functional *FRI* (FRI/FLC vs FRI/flc) | | | | | | | | | | | | | | | |
| Col-*FRI*_Sf_ | Col-*FRI*_Sf_ *flc-3* | WL | D | 0.17 |  | 1.03 |  | 14.04 | * | 0.34 |  | 2.24 |  | 0 |  |
|  |  |  | WL | 3.43 |  | 0.34 |  | 3.06 |  | 0.41 |  | 2.45 |  | 0.25 |  |
|  |  |  | NF | 4.4 |  | 0.02 |  | 0.99 |  | 9.29 |  | 1.88 |  | 5.25 |  |
|  |  |  | GF | 3.79 |  | 3.68 |  | 13.33 | * | 12.69 | * | 0.01 |  | 0.11 |  |
|  |  | NF | D | ~0 |  | 0.69 |  | 0.03 |  | 2.78 |  | 0 |  | 0 |  |
|  |  |  | WL | 0.37 |  | 3.36 |  | 0.86 |  | 0.17 |  | 5.8 |  | 1.61 |  |
|  |  |  | NF | 5.4 |  | 4.81 |  | 3.48 |  | 0.85 |  | 13.54 | * | 0.38 |  |
|  |  |  | GF | 3.58 |  | 13.91 | * | 10.87 |  | 12.23 |  | 1.17 |  | 0.59 |  |
|  |  | GF | D | 14.04 | * | 12.65 | * | 3.69 |  | 22.66 | *** | 0 |  | 2.33 |  |
|  |  |  | WL | 18.47 | ** | 53.26 | *** | 6.1 |  | 13.64 | * | 0.86 |  | 3.21 |  |
|  |  |  | NF | 13.68 | * | 1.75 |  | 0.33 |  | 0.04 |  | 0.05 |  | 0.08 |  |
|  |  |  | GF | 12.73 | * | 15.78 | ** | 1.85 |  | 1.15 |  | 0.57 |  | 0.09 |  |
| *FRI* disruption on non-functional *FLC* (FRI/flc vs fri/flc) | | | | | | | | | | | | | | | |
| Col-*FRI*_Sf_ *flc-3* | *flc-3* | WL | D | 28.6 | *** | 16.45 | ** | 2.64 |  | 2.78 |  | 0 |  | 0 |  |
|  |  |  | WL | 2.17 |  | 1.37 |  | 0 |  | 39.81 | *** | 0.03 |  | 8.91 |  |
|  |  |  | NF | 2.33 |  | 0.65 |  | 0.08 |  | 17.87 | ** | 4.13 |  | 4.77 |  |
|  |  |  | GF | 6.64 |  | 17.58 | ** | 0.7 |  | 10.08 |  | 0.05 |  | 0.23 |  |
|  |  | NF | D | 1.73 |  | 0.85 |  | 14.56 | * | 4.18 |  | 0 |  | 0 |  |
|  |  |  | WL | 1.74 |  | 1.52 |  | 0.01 |  | 0.07 |  | 1.87 |  | 0.43 |  |
|  |  |  | NF | 17.35 | ** | 1.98 |  | 6.27 |  | 1.88 |  | 4.45 |  | 1.94 |  |
|  |  |  | GF | 6.03 |  | 6.21 |  | 1.88 |  | 5.36 |  | 3.25 |  | 0.02 |  |
|  |  | GF | D | 0.34 |  | 0 |  | 2.95 |  | 12.58 | * | 0 |  | 0 |  |
|  |  |  | WL | 0.93 |  | 3.86 |  | 3.08 |  | 0.53 |  | 3.57 |  | 0.07 |  |
|  |  |  | NF | 0.02 |  | 5.88 |  | 6.02 |  | 0.32 |  | 0.43 |  | 1.45 |  |
|  |  |  | GF | 1.22 |  | 1.63 |  | 0.01 |  | 9.49 |  | 0.3 |  | 0.33 |  |

**Figure S1.** Experimental design. Diagram of different maturation light, imbibition light, imbibition temperature and after-ripening/secondary-dormancy treatments used in this study. “Comparisons” indicate the contrasts and their interpretation.

**Figure S2.** Effect of maturation under white light (WL), a neutral filter (NF), and a green filter (GF) (*x*-axis) on germination of fresh seeds, after-ripened seeds, and seeds induced into secondary dormancy of the L*er,* L*er-FLC* and Col genotypes (see key) kept in darkness, and at either 10°C (upper panel) or 22°C (lower panel). For statistical significance of pairwise comparisons (between maternal and seed imbibition conditions), see Table S2.

**Figure S3.** Direction and strength of the effect of seed maturation and imbibition under different light conditions at 10°C or 22°C for the L*er* genotype. Comparisons of WL vs NF, WL vs GF, and NF vs GF were made to assess the strength and direction of the maternal light environment of seeds incubated in WL (“Maternal light”, gray symbols), and of the imbibition light environment of seeds matured under WL (“Imbibition light”, black symbols). Rows indicate effects for fresh (upper row) or after-ripened seeds (lower row). Each value is the change in log odds with associated 97.5% confidence intervals of germination caused by changes in light environment during maturation and imbibition: positive values indicate that each environment (in column order: NF, GF, and GF) increases germination compared to the reference environment (in column order: WL, WL, and NF). Confidence intervals that cross zero (vertical dashed gray line) indicate there was no effect of the environment.

**Figure S4.** Direction and strength of the effect of seed maturation and imbibition under different light conditions at 10°C or 22°C for the L*er-FLC* genotype. Comparisons of WL vs NF, WL vs GF, and NF vs GF were made to assess the strength and direction of the maternal light environment of seeds incubated in WL (“Maternal light”, gray symbols), and of the imbibition light environment of seeds matured under WL (“Imbibition light”, black symbols). Rows indicate effects for fresh (upper row) or after-ripened seeds (lower row). Each value is the change in log odds with associated 97.5% confidence intervals of germination caused by changes in light environment during maturation and imbibition: positive values indicate that each environment (in column order: NF, GF and GF) increases germination compared to the reference environment (in column order: WL, WL and NF). Confidence intervals that cross zero (vertical dashed gray line) indicate there was no effect of the environment.

**Figure S5.** Direction and strength of the effect of seed maturation and imbibition under different light conditions at 10°C or 22°C for the Col genotype. Comparisons of WL vs NF, WL vs GF, and NF vs GF were made to assess the strength and direction of the maternal light environment of seeds incubated in WL (“Maternal light”, gray symbols), and of the imbibition light environment of seeds matured under WL (“Imbibition light”, black symbols). Rows indicate effects for fresh (upper row) or after-ripened seeds (lower row). Each value is the change in log odds with associated 97.5% confidence intervals of germination caused by changes in light environment during maturation and imbibition: positive values indicate that each environment (in column order: NF, GF, and GF) increases germination compared to the reference environment (in column order: WL, WL, and NF). Confidence intervals that cross zero (vertical dashed gray line) indicate there was no effect of the environment.

**Figure S6.** Effects of *FLC* activity on germination responses to light of genotypes in the L*er* background. Effect of maturation under white light (WL), a neutral filter (NF), and a green filter (GF) (*x*-axes) on germination of fresh seeds, after-ripened seeds, and seeds induced into secondary dormancy (rows) of genotypes in L*er* background—L*er*, *Ler-FLC*, L*er-FLC* RNAi-FLC #1 (RNAi #1) and RNAi #2—imbibed under WL, NF, or GF, or kept in darkness (D) (columns), and at either 10°C (a) or 22°C (b). See Table S1 for information on the genotypes and the Supplementary Text for a discussion of these results.

**Figure S7.** Effects of *FLC* activity on germination responses to light of genotypes in Col background. Effect of maturation under white light (WL), a neutral filter (NF), and a green filter (GF) (*x*-axes) on germination of fresh seeds, after-ripened seeds, and seeds induced into secondary dormancy (rows) of genotypes in Col background—Col (fri/FLC), Col-*FRI_Sf_* (FRI/FLC), Col-*FRI_Sf_ flc-3* (FRI/flc), and *flc-3* (fri/flc)—imbibed under WL, NF, or GF, or kept in darkness (D) (columns), and at either 10°C (a) or 22°C (b). See Table S1 for information on the genotypes and the Supplementary Text for a discussion of these results.
